# Active landslides on the Moon

**DOI:** 10.1093/nsr/nwaf384

**Published:** 2025-09-11

**Authors:** Zhiyong Xiao, Zhouxuan Xiao, Wuming Zhang, Shubing Ouyang, Yichen Wang, Yiren Chang, Hanxing Ouyang, Senmiao Wang, Jun Cui

**Affiliations:** Planetary Environmental and Astrobiological Research Laboratory, School of Atmospheric Sciences, Sun Yat‐sen University, Zhuhai 519082, China; School of Geospatial Engineering and Science, Sun Yat‐sen University, Zhuhai 519082, China; School of Geospatial Engineering and Science, Sun Yat‐sen University, Zhuhai 519082, China; Academy of Digital China (Fujian), Fuzhou University, Fuzhou 350003, China; Planetary Environmental and Astrobiological Research Laboratory, School of Atmospheric Sciences, Sun Yat‐sen University, Zhuhai 519082, China; Mathematics and Science College, Shanghai Normal University, Shanghai 200234, China; Planetary Environmental and Astrobiological Research Laboratory, School of Atmospheric Sciences, Sun Yat‐sen University, Zhuhai 519082, China; Planetary Environmental and Astrobiological Research Laboratory, School of Atmospheric Sciences, Sun Yat‐sen University, Zhuhai 519082, China; Planetary Environmental and Astrobiological Research Laboratory, School of Atmospheric Sciences, Sun Yat‐sen University, Zhuhai 519082, China

**Keywords:** Moon, moonquakes, landslides, impact craters, geohazard

## Abstract

Mass wasting of slope materials is a fundamental surface process on the Moon, yet its current activity and geohazard risks remain unconstrained. Here, we analyse multi-temporal images for terrains representing the least stable areas on the Moon, revealing new landslides formed in the past 15 years. The new landslides are superficial and small in size, displacing materials that are significantly less than 10^5^ m^3^ in volume. With localized occurrences, new landslides may pose limited hazards to future surface explorations, except for slope-proximal facilities and operations. Without a clear genetic relationship with thermal weathering of exposed crystalline rocks, ∼29% of the new landslides were likely triggered by new impact events, but the efficiency is comparatively smaller than that of endogenic seismic activity. Most new landslides were likely induced by endogenic moonquakes and they display distinct spatial clustering in the east of the Imbrium Basin, implying heterogeneous distributions of seismic zones in the lunar interior.

## Teaser

Change detection conducted in the youngest, topographically steepest, and theoretically most unstable regions on the lunar surface revealed a large number of new landslides formed since 2009. Endogenic moonquakes rather than new impacts are the primary trigger, and the Imbrium basin may host an active seismic zone.

## INTRODUCTION

Driven by gravity, movements of slope materials are a ubiquitous geological process on terrestrial bodies across the solar system, causing fatal geohazards on Earth [1]. On the surfaces of single-plate planetary bodies where endogenic geological activity has been sluggish, such as the Moon, mass wasting is one of the most important agents of modifying surface topography [2]. Landslide deposits are pervasive on the Moon and they have various dimensions and emplacement mechanisms, such as meter-scale regolith clumps formed by soil creeping on shallow slopes, fast rockfalls and granular flows and slumps of crater walls [2–5]. Among potential triggers of lunar landslides, collisions by extralunar impactors and endogenic seismic activity were interpreted to play the dominating role [2, 3, 5, 6], while thermal weathering of exposed crystalline rocks might be an additional cause [7].

Age estimations based on crosscutting relationships and crater statistics revealed that late Copernican landslides are widespread on the Moon [2, 3, 5]. On the basis of high-resolution images that were obtained under similar illumination conditions but at different times (i.e. temporal images), a global survey of short-term changes in the last decade yielded thousands of new impacts [8] and a dozen landslides [9], suggesting that, currently, impacts are the dominating active surface process and mass wasting is ongoing on the Moon [8,9]. Investigations for the geological context of the new landslides suggested that both new impacts and moonquakes formed by fault slip are potential triggers [10], but their relative contribution remains unclear. Meanwhile, besides recent tectonism [11,12], magma activity may have lasted to ∼100 Ma on the Moon [13,14] and they both serve as potential sources of recent moonquakes.

Lunar exploration is accelerating. Human civilization has never been closer to establishing permanent infrastructures on the Moon, which will serve as scientific research stations and/or deep-space outposts [15]. While moonquakes were detected during the Apollo missions [cf. 2], conventional geological wisdom posited that lunar endogenic activity had essentially ceased [14], leaving geological hazard assessments of lunar seismicity largely unexplored. On terrestrial planets, landslides are a direct consequence of seismic activity [1], yet the current activity level of lunar landslides remains poorly constrained and the geohazard potential of active landslides for future lunar surface missions has not been systematically evaluated.

## RESULTS

### Morphology and geometry of new landslides

**Figure 1.**
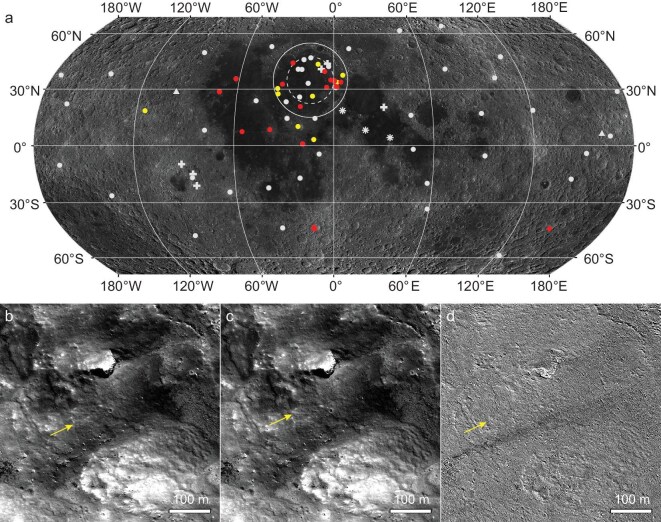
Observation targets and an example of new landslide on the Moon. (a) Locations of the 74 observation targets on the Moon that are classified into 7 types. Yellow and red dots depict new landslides that are potentially triggered by new impacts (Table S1) and endogenic seismic activity (Table S2), respectively. Gray dots are young craters inspected, including Erathosthenian- and Copernican-aged complex craters [36], cold spot craters [26] and new craters >28 m in diameter [23]. Gray crosses, stars and triangles denote the inspected young wrinkle ridges, possible epicenters of strong shallow moonquakes [19,29] and irregular-mare patches [13], respectively. White dashed and solid circles depict rims of the transient cavity and the final topographic rim of the Imbrium Basin, which have diameters of ∼721 and ∼1160 km, respectively [32]. The information of temporal images inspected is shown in the Data S1. (b–d) Before, after and temporal ratio images of a new landslide (centered at 43.338°S, 13.025°W) on the western wall of the Tycho crater, respectively. New impacts and exposed crystalline rocks are not obvious in the initiation zone of this landslide, suggesting that the landslide was likely triggered by endogenic seismic activity. Yellow arrows point to downslope directions (39°; Table S2). IDs and addresses of data used in this figure are available in Table S3.

Using high-resolution temporal images obtained by the Narrow Angle Camera of the Lunar Reconnaissance Orbiter Camera (LROC NAC) [16], we detected new landslides on the Moon that have occurred since 2009. The precise co-registration of before and after images is critical for detecting short-term surface changes and identifying potential landslides [8]. However, matching feature points in temporal image pairs of lunar slope areas remains challenging due to complex local illumination conditions, necessitating frequent manual adjustment (see ‘Materials and methods’). On the other hand, landslides on planetary bodies preferentially occur on steep slopes mantled by unconsolidated debris [2] and in geologically active terrains [3]. On the Moon, crater walls and central peaks of young impact craters often host debris-covered steep slopes that have angles approaching the repose angle of lunar terrains (∼32°) [17,18]. Regions potentially hosting active endogenic geological processes are also widespread on the Moon, such as wrinkle ridges formed by fault movements in the late Copernican [11,12,19,20] and irregular-shaped mare patches that might be related to recent magma activity [13]. In this work, we select typical young craters, tectonic features and irregular-shaped mare patches to search for short-term changes that occurred in the past 15 years (Data S1). In total, 562 pairs of temporal images (Data S1) across 74 observation targets (7 types) are manually co-registered and inspected (Fig. 1a). The investigated terrains are among the most active and least stable areas across the globe and each terrain type contains a comparable number of observation targets at the nearside and farside (Data S1 and Fig. 1a). Therefore, the occurrences and spatial distribution of new landslides on these terrains are largely representative of the current activity level of landslides on the Moon.

In addition to abundant new impacts (Fig. S1), 41 new landslides are confidently identified, which are comparable to the number of new landslides discovered in an earlier global search [9]. This comparison further suggests that landslides on the Moon preferentially occur in unstable regions. The new landslides are mostly <1 km long and <100 m wide (Figs S2–S34). Initiated from local high elevations, new landslides occur as superficial disturbances on terrains with slope angles of 24°–42° (Tables S1 and S2). Possibly related to diverging particle sizes and/or packing conditions, the landslide deposits predominantly exhibit lower reflectances than the underlying materials (e.g. Figs S2–S32), but one exceptional case shows higher reflectances (e.g. Fig. S33). Materials displaced by the landslides are barely discernible in the after images and neither accumulation zones nor topographic margins are visible as seen in sub-pixel-scale imagery (Fig. 1c and Figs S2–S34), suggesting that the landslide deposits have thicknesses smaller than pixel scales of LROC NAC images. In fact, the majority of the landslide deposits are only obvious as seen in the temporal ratio images (Fig. 1d and Figs S2–S34). Therefore, the landslide deposits are inferred to be <1 m thick, yielding displaced mass volumes that are significantly less than 10^5^ m^3^ for each case.

High-resolution imagery (≤1 m/pixel) reveals no topographic depressions in the initiation zones of the new landslides (Fig. 1b and Figs S2–S34). Assuming that most of the displaced materials (e.g. with a volume of tens to hundreds of m^3^) originated from the initiation zones, cavities left by mass movements should be resolvable with this image resolution. Therefore, initiation of the new landslides involved materials with much smaller volumes than the image resolution, suggesting that most displaced materials may consist of locally disturbed fine debris along the movement paths, rather than materials displaced from upslopes. Compared with terrestrial landslides [21], the new lunar landslides are small-scale and localized mass wasting phenomena, indicating relatively low geohazard potential for future lunar surface explorations, except for slope-proximal facilities and operations.

### Landslides triggered by new impacts

**Figure 2.**
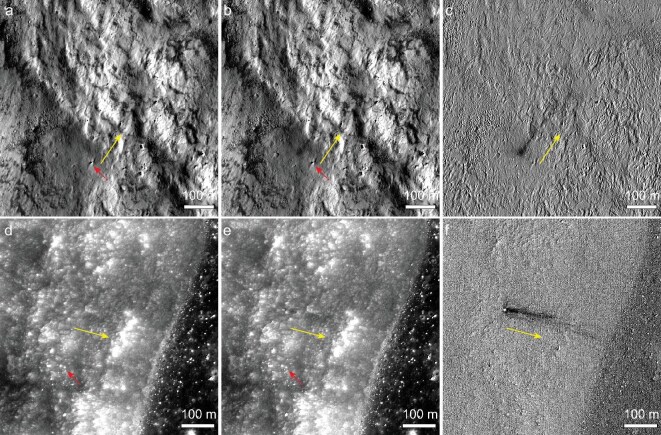
New landslides that may be directly triggered by new impacts. (a–c) and (d–f) are before, after and temporal ratio images of a new landslide on the southwestern wall of the Diophantus (centered at 27.420°N, 34.451°W) and western wall of the Copernicus crater (centered at 10.187°N, 21.473°W), respectively. The two image pairs were obtained with a time interval of <1 year (Table S1). Red dashed arrows point to meter-sized hanging boulders near the impact sites. Yellow arrows point to downslope directions. New impacts are visible at the immediate heads of the landslides and they occur as splotches caused by impact rays [8], while the topography of impact craters is not discernible due to their small sizes. IDs and addresses of data used in this figure are available in Table S3.

In total, 29% (*n* = 12/41) of the detected landslides exhibit spatial association with new impact events right in their initiation zones (Figs S2–S11). The new impacts have the diagnostic morphology of impact rays that occur as superficial splotches, which were caused by subtle changes in ray reflectances [8] (Fig. 2). Rim crests of impact craters are not discernible in the splotches (Fig. 2b and e), suggesting that their diameters are smaller than ∼2 pixels of the base images [22], i.e. <1–2 m. For such cases, some of the temporal images were acquired with a time interval of <1 year (Fig. 2 and Table S1), suggesting that the new landslides might be directly triggered by the new impacts, e.g., via impact-induced strong ground shaking. However, albeit a close spatial association, the time intervals of the available temporal images are usually not short enough to definitively establish synchroneity between the new impacts and landslides (Data S1).

The new landslides that were likely triggered by new impacts occur immediately downslope from the impact points (Fig. 2 and Figs S2–S11), with sizes comparable to those lacking new impacts in the initiation zones (e.g. Fig. 1d). Background terrains of impact-induced landslides predominantly have slope angles of >31° (Table S1), approaching the repose angle of lunar terrains [17]. Therefore, the pre-landslide slope materials may have already been in a quasi-stable state before the new impacts occurred. Adjacent to impact-induced landslides, meter-sized hanging boulders show no evidence of rockfalls (Fig. 2), indicating limited disturbances to slope materials proximal to the impact sites.

### Limited efficiency of impacts in triggering landslides

**Figure 3.**
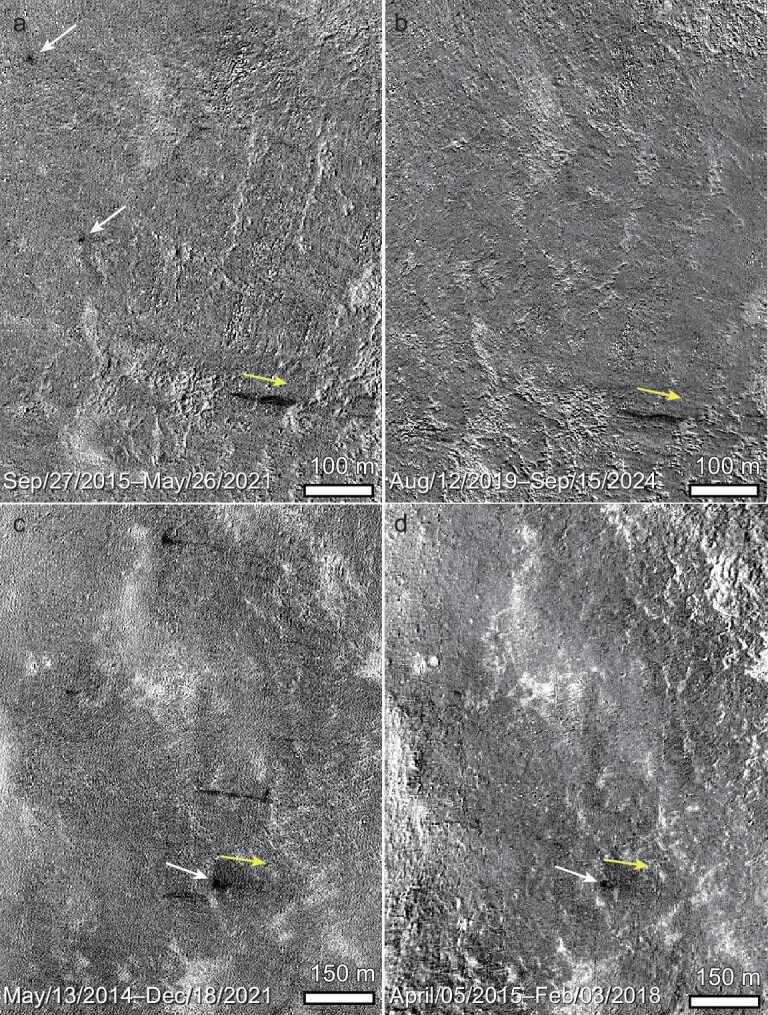
Temporal ratio images obtained at different time intervals revealing the different formation times of new impacts and landslides on the same slope. (a, b) Two new impacts on the western crater wall of Heis (centered at 32.555°N, 32.216°W) occurred between 12 August 2019 and 26 May 2021, later than the new landslide. (c, d) New impact on the western crater walls of Naumann (centered at 35.385°N, 62.184°W), denoted by the white arrow, formed earlier than the new landslide in the surroundings. Yellow arrows point to downslope directions. IDs and addresses of data used in this figure are available in Table S3.

Occasionally, new impacts and landslides co-occur on the same slope, but the new impacts do not occur in the initiation zones of the landslides, as they are spatially separated by distances of up to ∼500 m (Fig. 3a and c). By tracking their separated formation times using temporal images that have progressively smaller intervals of acquisition times, we found that the new impacts and the adjacent landslides were temporally independent, suggesting that they have no genetic linkage (Fig. 3b and d).

**Figure 4.**
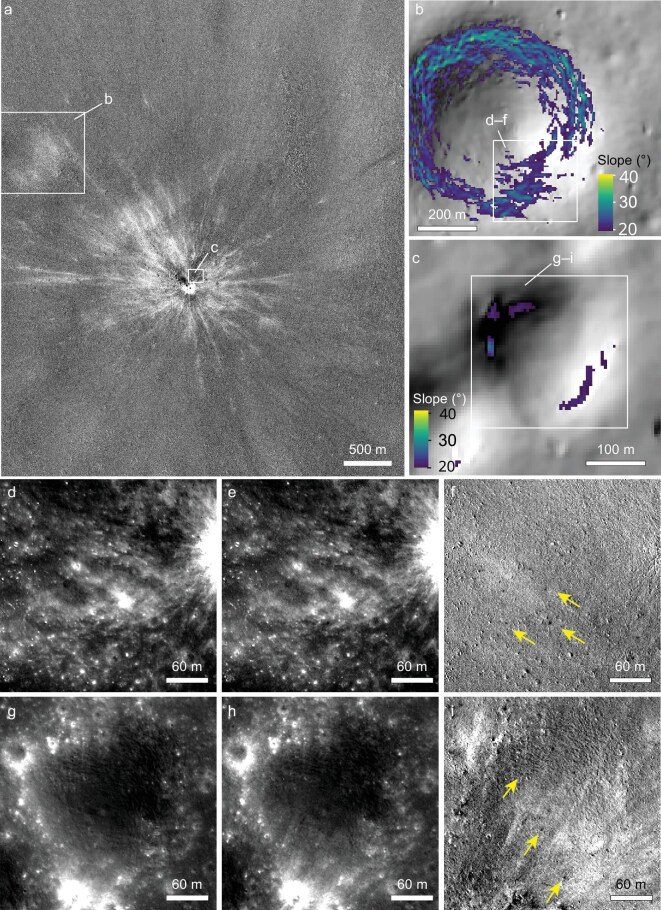
Low efficiency of impact cratering and ejecta deposition in triggering landslides. (a) Temporal ratio image shows surface changes caused by a new impact crater (central coordinates 17.167°S, 20.041°W) that has a diameter of 28 m [23]. (b–c) Steep slopes of background terrains of the new crater (locations shown in panel (a)). (d–f) and (g–i) are before, after and temporal ratio images of secondary impact splotches and impact rays on steep slopes of the background terrain (yellow arrows), respectively. Changes in reflectances caused by impact rays and secondary impacts are abundant on slopes, but no new landslides are visible. Note that impact rays on slopes have radial distribution about the parent crater and they do not follow local downslopes as landslides do (i). Slope analysis was conducted using bidirectional slopes at a 5-m grid resolution. Locations of panels (d–f) and (g–i) are shown in panels (b) and (c), respectively. IDs and addresses of data used in this figure are available in Table S3.

In addition, impacts in the past 15 years on the Moon have formed craters of up to ∼75 m in diameter [23], producing widespread impact rays and secondary impacts as far as >30 km from the parent craters [8,22]. However, possible new landslides triggered by the relatively large new impacts have not yet been reported. The new impact shown in Fig. 4 is among the largest to have formed in the past 15 years [23]. Around this crater, surrounding terrains with slope angles of >20° contain abundant impact rays and secondary impact splotches (Fig. 4a–c). However, impact-induced seismic waves and ejecta deposition did not induce landslides on these steep slopes (Fig. 4d–i). This observation indicates that, while new impacts could induce landslides via ground shaking on immediate downslopes (Fig. 2), seismic waves formed by the primary impacts and disturbances caused by the landing of their ejecta are inefficient to induce landslides beyond the impact sites. This finding aligns with the minor portion of potential impact-induced landslides in our catalog (Tables S1 and S2). It is also consistent with an earlier global search of short-term changes on the Moon, which are predominantly caused by new impacts, but new landslides are orders of magnitude less frequent [9].

### Landslides triggered by endogenic seismic activity

The majority of the detected landslides (71%; *n* = 29/41) show no spatial association with new impacts (Figs 1d and 3) and their background slope angles are comparable to those of impact-triggered landslides (Table S2). The host craters are predominantly pre-Copernican (69%; *n* = 11/16; Table S2), indicating that both the exposure age and the local slope of the background terrains are secondary factors affecting slope material stability. On the other hand, it has not yet been confirmed whether or not diurnal temperature changes on the Moon could break down surface materials, but exposed crystalline rocks should be more prone to thermal weathering, as numerical modeling shows that a superficial cover of regolith would efficiently insulate temperature changes in underlying materials [7]. In the before images used to detect short-term changes, however, exposed crystalline rocks are not visible in the initiation zones of the new landslides (Figs S12–S34). Therefore, new landslides that are not associated with new impacts in their heads were not obviously induced by breakdown of bedrocks caused by thermal weathering.

Among known triggers of landslides on the Moon [2, 3, 5–7], endogenic seismic activity is the most plausible cause of most of the detected new landslides. While sources of endogenic seismic activity on the Moon are not yet completely understood [20], this interpretation aligns with the current thermal state of the Moon—that the lunar interior remains active enough to drive endogenic seismic activity [11,20]. New landslides that were likely triggered by endogenic seismic activity are predominantly located on the nearside mare (Fig. 1a). While the statistical significance of this spatial clustering requires further validation by using a global inventory of active landslides, the first-order distribution is considered reliable, because the 75 locations examined are likely representative for the current activity level of landslides on the Moon and the clustered distribution is consistent with the concentrated occurrences of endogenic shallow moonquakes at the northern hemisphere of the lunar nearside [20].

In addition, new landslides possibly triggered by endogenic seismic activity exhibit distinct clustering between the transient cavity and the rim crest of the Imbrium Basin, in sharp contrast to the absence of landslides in the eastern Orientale Basin (Fig. 1a). In the eastern Imbrium Basin, multiple landslides are visible on the same walls of impact craters, such as the northwestern wall of Aristillus (Figs S13–S17 and S35) and Gambart A craters (Figs S24 and S25). However, the temporal resolution of available temporal images is insufficient to confirm whether these multiple landslides formed at different times—a critical indicator of prolonged and localized seismic activity. For comparison, our inspection shows that not all craters in this region host active landslides (Fig. 1a), indicating that the epicenters of endogenic moonquakes might be highly nonuniform in this region.

## DISCUSSION

Synthesis of observations suggests that endogenic seismic activity may be the primary driver of contemporary landslides on the Moon. If the thermal weathering of exposed bedrocks or new impacts were the dominant triggers, then landslides would be equally or more abundant on the steep slopes of young impact craters, where exposures of fresh bedrocks and steep slopes prevail. Contrarily, the observed landslides do not preferentially occur in Copernican-aged craters, such as Aristarchus, Jackson and Glusko (Fig. 1a). Change detection on the walls of much younger craters did not yield positive identification of new landslides either, despite their wall slopes of >20° [18,23]. For example, Figs S36–S41 show negative detection in the Giordano Bruno crater that formed at ∼4 Ma [24], cold spot craters that are younger than ∼1 Ma and formed widespread ray structures with lower night temperatures than mature regolith [25–27] and new craters formed in the past 15 years [23]. Therefore, while pristine impact craters are the least stable regions on the Moon from the perspective of topographic degradation at geological timescales [28], they exhibit short-term stability at the timescale of ≥15 years. These results challenge existing models for triggers of lunar landslides, advocating the systematic re-evaluation of thermal weathering and impact-induced disturbances as triggering mechanisms [2–7,10]. Using the geological thought of extrapolating the past from the present (i.e. uniformitarianism), ancient landslides on the Moon may also be mainly triggered by endogenic moonquakes, although impact cratering and thermal weathering have contributed to the breakdown of bedrocks and possibly triggered a minor portion of landslides.

Among the widespread young wrinkle ridges on the Moon that were formed by thrust faults, some were interpreted to be the epicenters of strong shallow moonquakes recorded by Apollo seismometers (Fig. 1a), which might have triggered rockfalls that were observed on the background terrains [19,29]. However, for these wrinkle ridges and their adjacent terrains, our inspection did not detect new landslides, but did discover abundant new impacts (Figs S42–S46). This observation suggests that the investigated faults may not have been active enough in the past 15 years or that the Apollo shallow moonquakes were induced by slips of the other fault segments [20]. Similarly, change detection on irregular-mare patches, such as Ina, did not discover new landslides either, but new impacts are also abundant there (Figs S47–S49). Therefore, if fault movements and/or magma activity in these regions had induced seismic activity in the past 15 years, then the seismic magnitudes may be smaller than those in the Imbrium Basin.

Formed ∼3.92 Ga [30], the Imbrium Basin is one of the most active areas on the Moon in terms of structural deformation, as the region between its transient cavity and rim crest hosts the active nearside tectonic system [31]. Here, meter-sized boulders exposed on wrinkle ridges and lobate scarps are believed to be replenished due to fault reactivation, which may be driven by deep stresses imposed by the antipodal impact that formed the ancient South Pole-Aitken Basin [31]. Therefore, the apparent clustered spatial distribution of active landslides in the Imbrium Basin supports elevated moonquakes in this region. This interpretation is also consistent with the recent observation that the Apollo 15 landing site, which is located within the Imbrium Basin, may be more seismically active than the Apollo 14 and 16 landing sites [20]. Therefore, the Imbrium Basin, especially the eastern portion, will be ideal for deploying seismometers and thermal probes to characterize the thermal state and interior dynamics of the Moon. This study further proposes that the spatio-temporal distribution of active landslides triggered by endogenic moonquakes on the Moon can serve as proxies for subsurface seismic zones.

## MATERIALS AND METHODS

### Selection and process of temporal images

This study mainly uses experiment data records (EDRs) of images obtained by the NAC of the LROC [16]. Temporal image pairs consist of before and after images obtained at different times for the same area and with similar solar illumination directions. The maximum differences in incidence and emission angles are 5°, which are smaller than those used earlier for the detection of new impacts on the Moon [8]. This is because landslides on the Moon typically occur on steep mountains (e.g. wrinkle ridges) and topographic depressions (e.g. impact craters) and the complicated local topography and illumination conditions pose more of a challenge in the registration of before and after images compared with relatively smooth terrains. Therefore, by applying smaller differences of incidence and emission angles for the before and after images, false recognition can be minimized, although suitable temporal images are limited. For this reason, this study does not intend to perform a global search of new landslides, to avoid incomplete recognition. Nevertheless, 562 pairs of temporal images are manually inspected (Data S1) for the 74 observation targets (Fig. 1a), yielding a similar number of positive identifications of new landslides to an earlier global search of short-term changes on the Moon [9].

Preprocessing of the LROC NAC EDRs followed the standard pipeline by using the United States Geological Survey Integrated Software for Imagers and Spectrometers (ISIS3), including the following steps: attachment of camera position information, geometric calibration, radiometric calibration, echo correction and map projection (https://www.lroc.asu.edu/). To improve the precision of the correction and projection, the Selene-Lunar Orbiter Laser Altimeter digital elevation model (SLDEM) [33] and LROC NAC Digital Terrain Models (DTMs) [34] that have relatively high resolution are used.

### Registration of temporal images and manual inspection for change detection

Precise co-registration of temporal images is the critical step to detect potential short-term changes. We adapt and improve the co-registration method used earlier for the detection of new impacts on the Moon [8]. We mainly use the automatic registration module *coreg* in ISIS3 to find feature points in before and after images. The sizes of the matching window (i.e. pattern chip) and search window (search chip) are manually adjusted for each pair of temporal images to evaluate the performance of co-registration, as matching feature points by using a uniform setting of window sizes is usually hindered by pervasive small shadows on rough terrains. Furthermore, manual intervention for the extraction of feature points is necessary to adjust and optimize the matching results. The need for human input for the co-registration of temporal images further prohibits this study from performing an automatic global search, which may contain incomplete and/or false identification of landslides, hindering reliable evaluation for their activity level.

We calculate the temporal ratio images for co-registered image pairs by dividing the after image by the before image. The pixel values of the ratio images are mostly close to 1 because the before and after images are for the same region and under similar illumination conditions. Pixels with abnormally large or small values may be caused by short-term changes, but the possibility of imperfect registration of the temporal pairs should be excluded first. Therefore, manual inspection was carried out on each of the 562 temporal images, recognizing landslides from the detected changes. This procedure is time-consuming but with high precision for positive recognition. We were aware that our manual inspection yielded better recognition of true and false changes but, at the same time, we lost overlapped pixels that did not have good co-registration. This shortage is acceptable for the purpose of this work because we are not intending to evaluate the occurrence rate of new landslides, which otherwise would need precise areas of well-registered overlapped pixels of the before and after images [8].

High-resolution LROC NAC DTMs in the LROC data repository covered only a few of the investigated targets [23]. Therefore, the terrain slopes of the observed new landslides are measured by using SLDEM with a baseline of 60 m (Table S1). In addition, images obtained by the Kaguya Terrain Camera [35] are used to aid the investigation of geological context for the inspected targets.

## Supplementary Material

nwaf384_Supplemental_Files
