# Supplementary material for: Active landslides on the Moon
Source: Natl Sci Rev. 2025 Sep 11;12(11):nwaf384. doi: 10.1093/nsr/nwaf384 (PMC12576953; doi:10.1093/nsr/nwaf384)
Supplement: nwaf384_Supplemental_Files [file nwaf384_supplemental_files.zip › 2025-434-supplementarymaterials.docx]

**Supplementary Information**

**Active landslides on the Moon**

Zhiyong Xiao^1^*, Zhouxuan Xiao^2^, Wuming Zhang^2^, Shubing Ouyang^3^, Yichen Wang^1^, Yiren Chang^4^, Hanxing Ouyang^1^, Senmiao Wang^1^, Jun Cui^1^

^1^Planetary Environmental and Astrobiological Research Laboratory, School of Atmospheric Sciences, Sun Yat‐sen University, Zhuhai, China, 519082.

^2^School of Geospatial Engineering and Science, Sun Yat‐sen University, Zhuhai, China, 519082.

^3^Academy of Digital China (Fujian), Fuzhou University, Fuzhou, China, 350003.

^4^Mathematics and Science College, Shanghai Normal University, Shanghai, China, 200234.

^*^Corresponding author. Email: xiaozhiyong@mail.sysu.edu.cn

**This file includes:**

Supplementary Figs S1 to S49

Supplementary Tables S1 to S3

References (i.e., 19, 23, 25, 29, 35 in the main text)


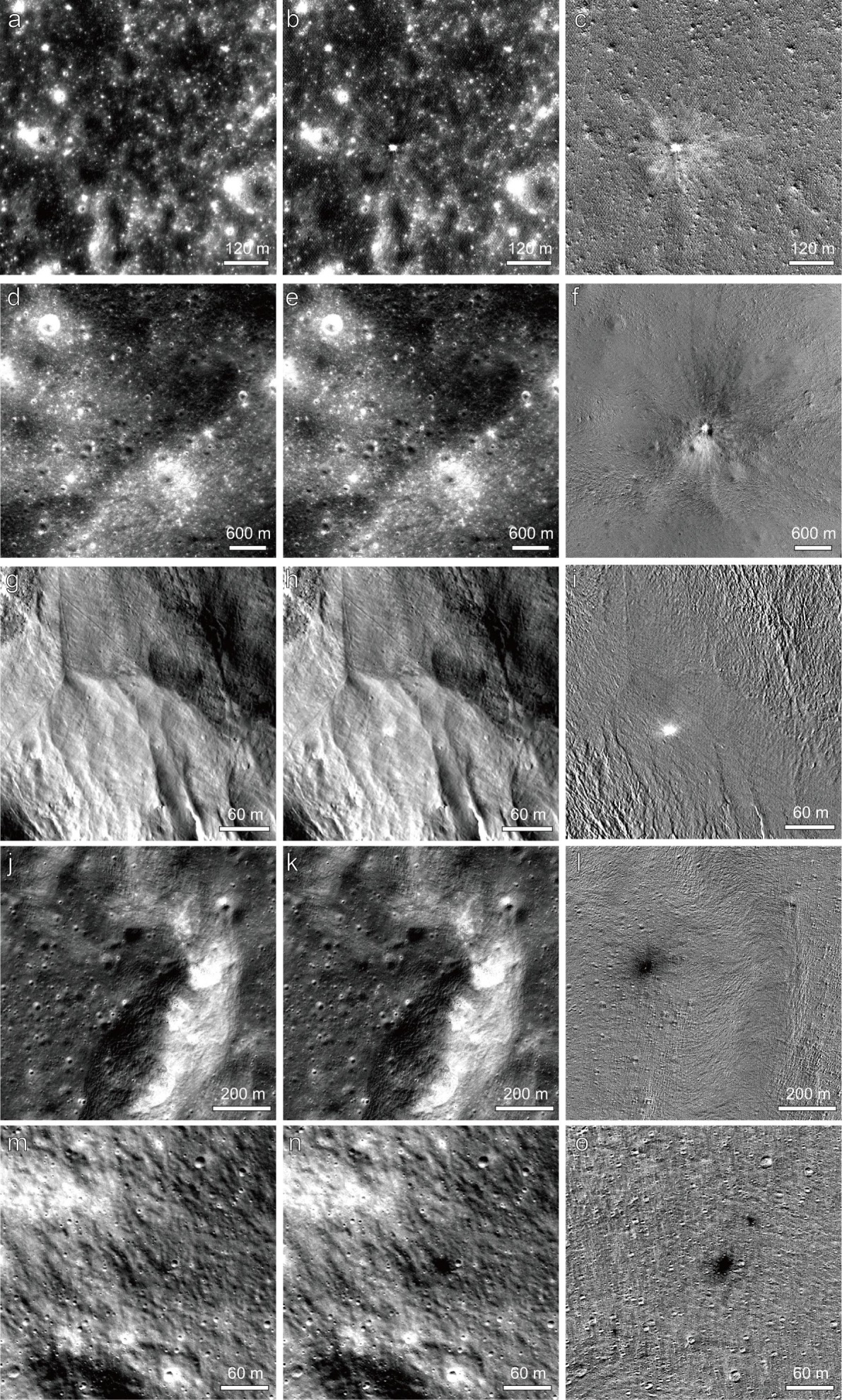


Supplementary Fig. S1. Examples of new impacts detected in this work. The first, second and third columns are for the before, after and temporal ratio images, respectively. The central coordinates of the five new impacts from top to bottom are:46.552°N, 4.852°W; 47.853°S, 94.76°W; 20.634°S, 70.927°W; 29.107°N, 98.380°W; and 9.013°N, 58.153°E, respectively. IDs and addresses of data used in this figure are available at Table S3.


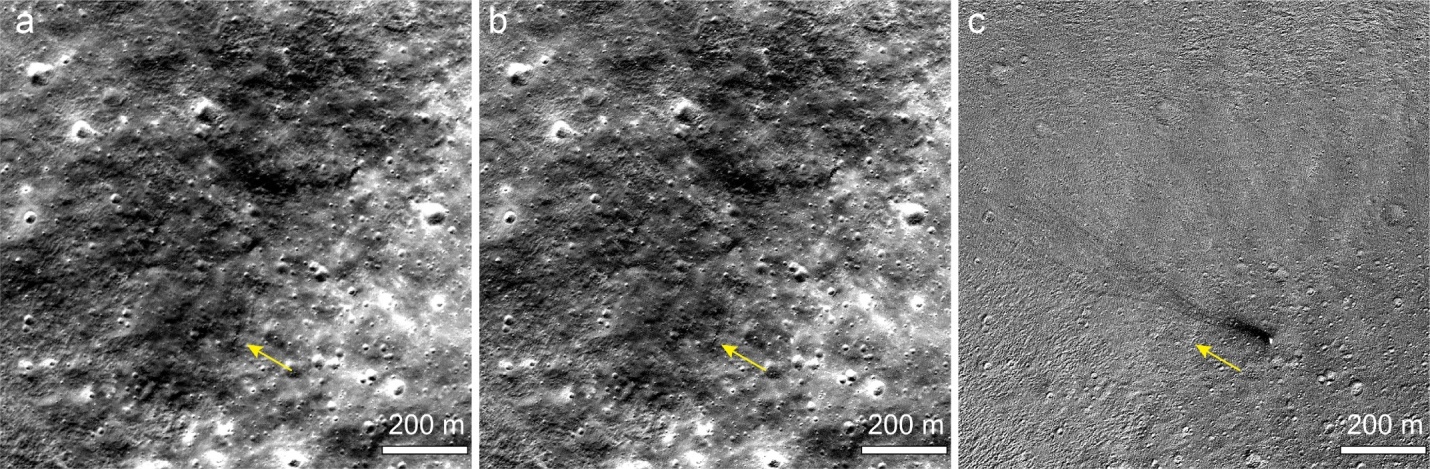


Supplementary Fig. S2. A new landslide that was likely triggered by a new impact on the southeastern crater wall of Aristillus. (a–c) Before, after and temporal ratio images, respectively. The coordinates of the new landslide are 33.491°N, 2.098°E. Yellow arrows point to downslope directions. The new impact is visible right in the initiation zone of the landslides, and it occurs as splotch caused by impact rays [8], while the topography of impact crater is not discernible due to its small size. IDs and addresses of data used in this figure are available at Table S3.


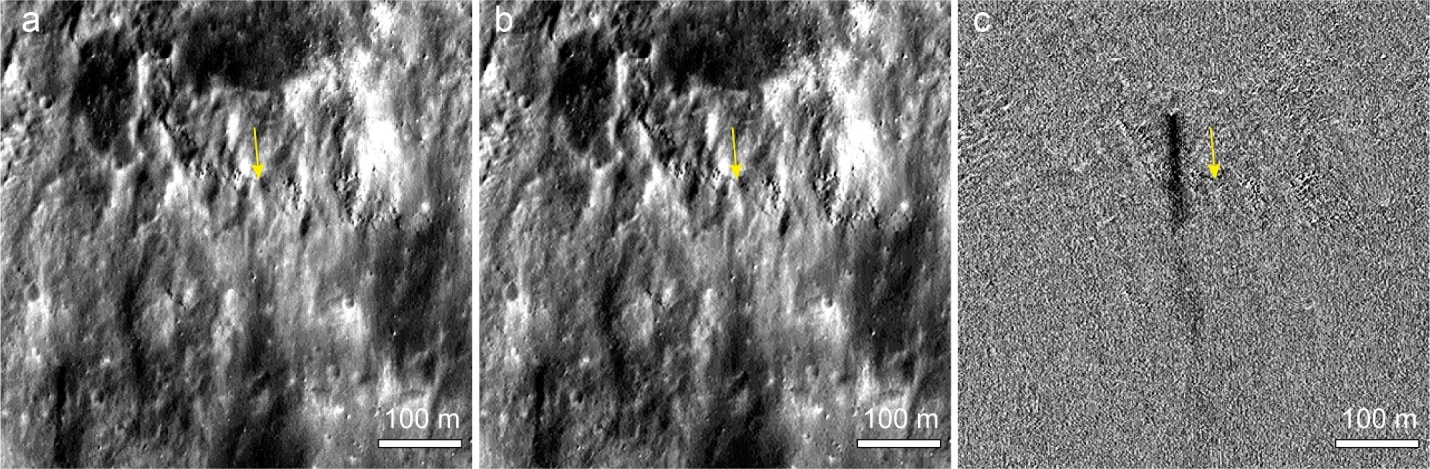


Supplementary Fig. S3. A new landslide that was likely triggered by a new impact on the northern crater wall of Briggs B. (a–c) Before, after and temporal ratio images, respectively. The coordinates of the new landslide are 28.535°N, 70.924°W. Yellow arrows point to downslope directions. The new impact is visible right in the initiation zone of the landslides, and it occurs as splotch caused by impact rays [8], while the topography of impact crater is not discernible due to its small size. IDs and addresses of data used in this figure are available at Table S3.


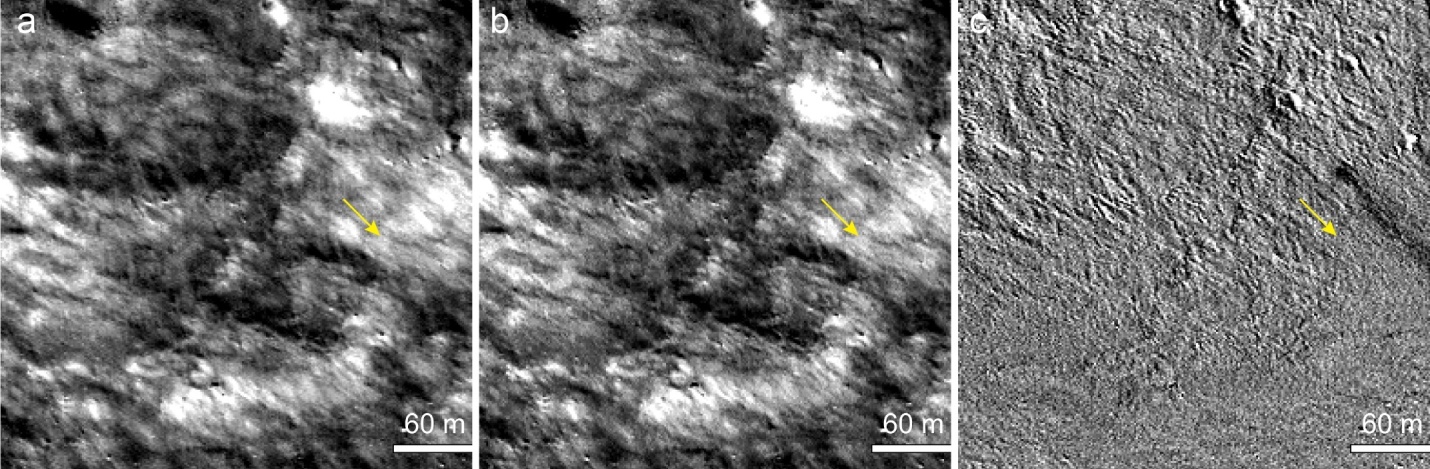


Supplementary Fig. S4. A new landslide that was likely triggered by a new impact on the northwestern crater wall of Delisle. (a–c) Before, after and temporal ratio images, respectively. The coordinates of the new landslide are 30.229°N, 34.946°W. Yellow arrows point to downslope directions. The new impact is visible right in the initiation zone of the landslides, and it occurs as splotch caused by impact rays [8], while the topography of impact crater is not discernible due to its small size. IDs and addresses of data used in this figure are available at Table S3.


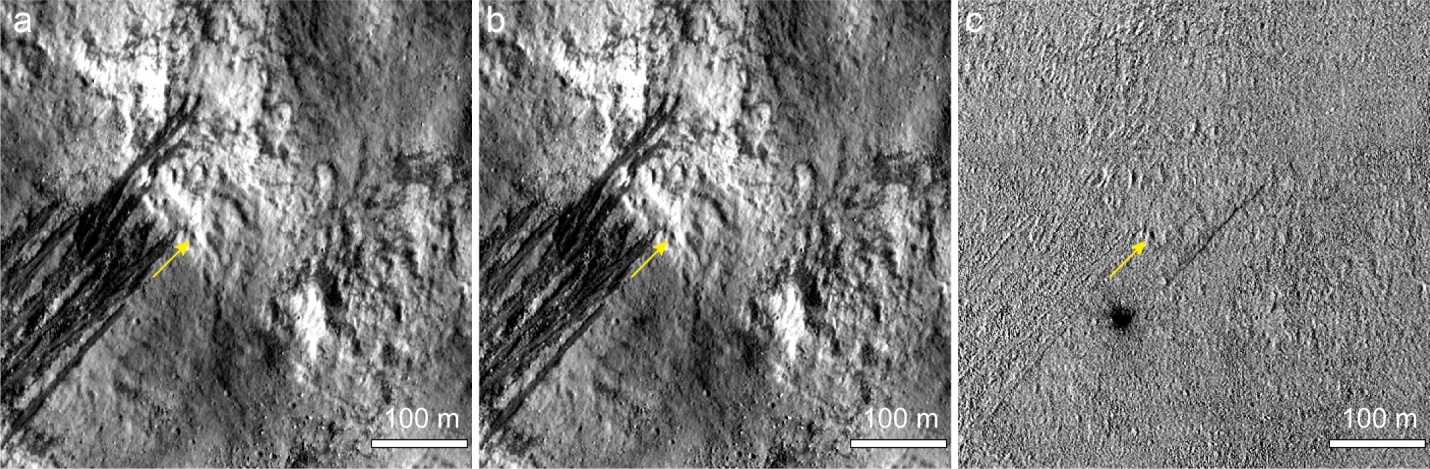


Supplementary Fig. S5. A new landslide that was likely triggered by a new impact on the southwestern crater wall of Gambart C. (a–c) Before, after and temporal ratio images, respectively. The coordinates of the new landslide are 3.218°N, 11.908°W. Yellow arrows point to downslope directions. The new impact is visible right in the initiation zone of the landslides, and it occurs as splotch caused by impact rays [8], while the topography of impact crater is not discernible due to its small size. IDs and addresses of data used in this figure are available at Table S3.


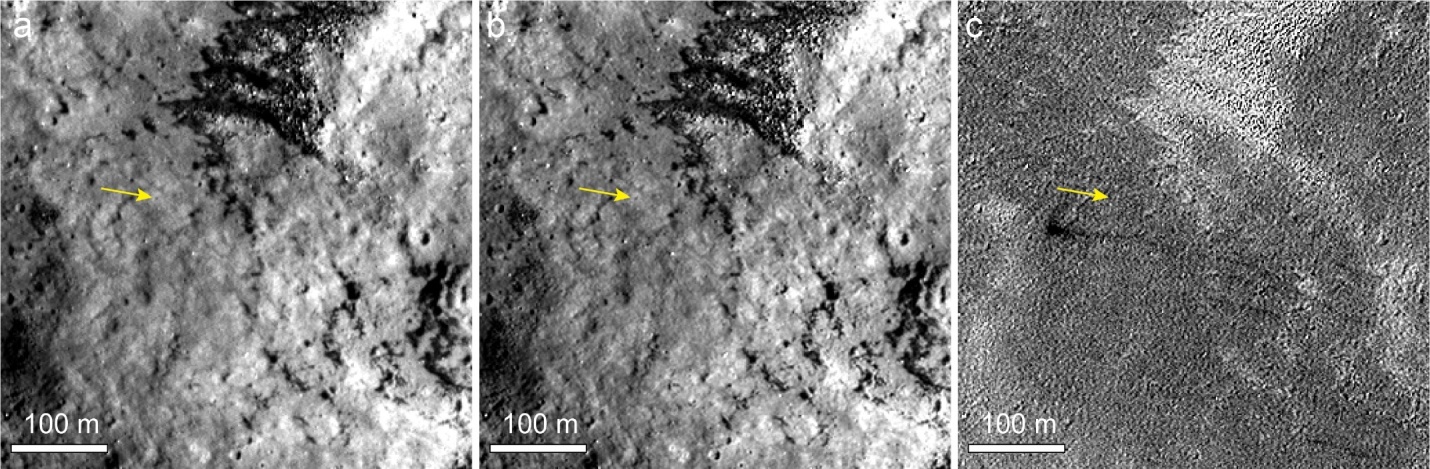


Supplementary Fig. S6. A new landslide that was likely triggered by a new impact on the western crater wall of Naumann. (a–c) Before, after and temporal ratio images, respectively. The coordinates of the new landslide are 35.41°N, 62.188°W. Yellow arrows point to downslope directions. The new impact is visible right in the initiation zone of the landslides, and it occurs as splotch caused by impact rays [8], while the topography of impact crater is not discernible due to its small size. IDs and addresses of data used in this figure are available at Table 3.


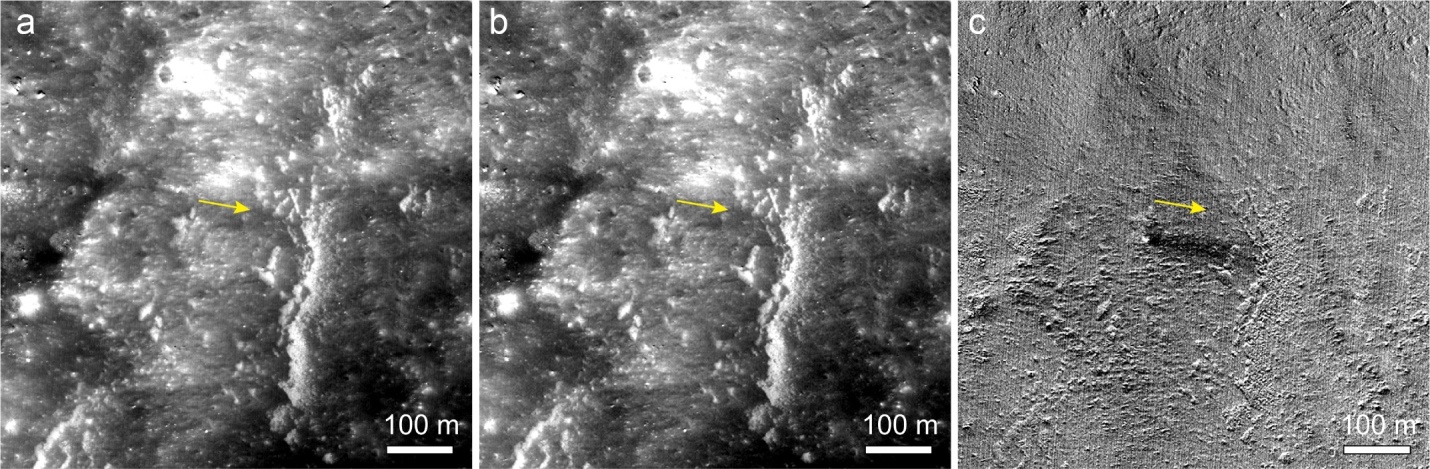


Supplementary Fig. S7. A new landslide that was likely triggered by a new impact on the eastern crater wall of Ohm. (a–c) Before, after and temporal ratio images, respectively. The coordinates of the new landslide are 18.542°N, 114.754°W. Yellow arrows point to downslope directions. The new impact is visible right in the initiation zone of the landslides, and it occurs as splotch caused by impact rays [8], while the topography of impact crater is not discernible due to its small size. IDs and addresses of data used in this figure are available at Table 3.


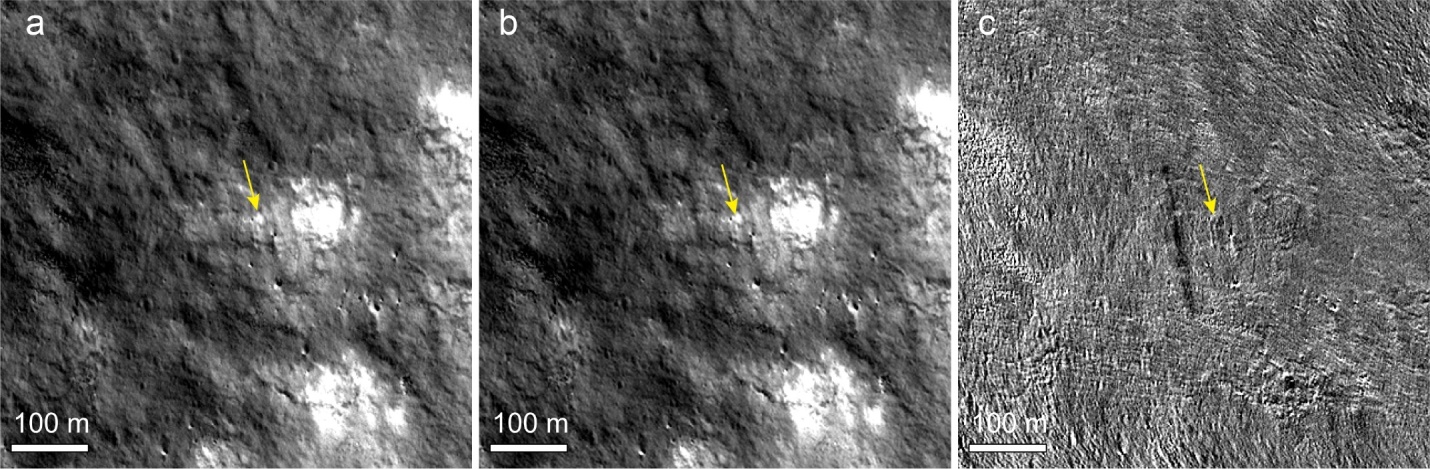


Supplementary Fig. S8. A new landslide that was likely triggered by a new impact on the northern crater wall of Pico E. (a–c) Before, after and temporal ratio images, respectively. The coordinates of the new landslide are 43.104°N, 10.334°E. Yellow arrows point to downslope directions. The new impact is visible right in the initiation zone of the landslides, and it occurs as splotch caused by impact rays [8], while the topography of impact crater is not discernible due to its small size. IDs and addresses of data used in this figure are available at Table 3.


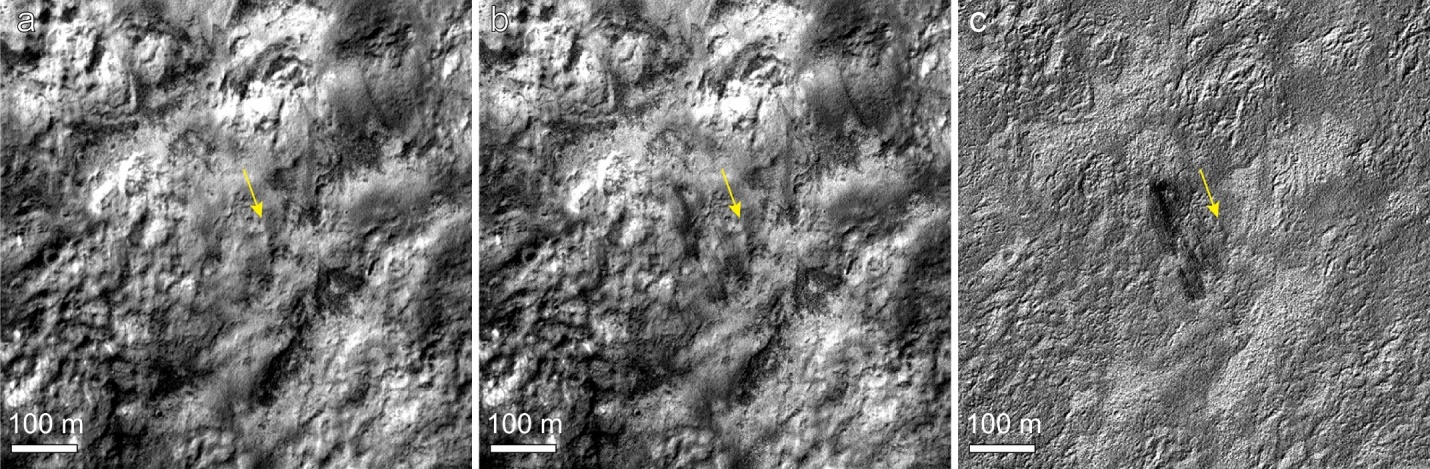


Supplementary Fig. S9. A new landslide that was likely triggered by a new impact on the northwestern crater wall of Theaetetu. (a–c) Before, after and temporal ratio images, respectively. The coordinates of the new landslide are 37.260°N, 5.799°E. Yellow arrows point to downslope directions. The new impact is visible right in the initiation zone of the landslides, and it occurs as splotch caused by impact rays [8], while the topography of impact crater is not discernible due to its small size. IDs and addresses of data used in this figure are available at Table 3.


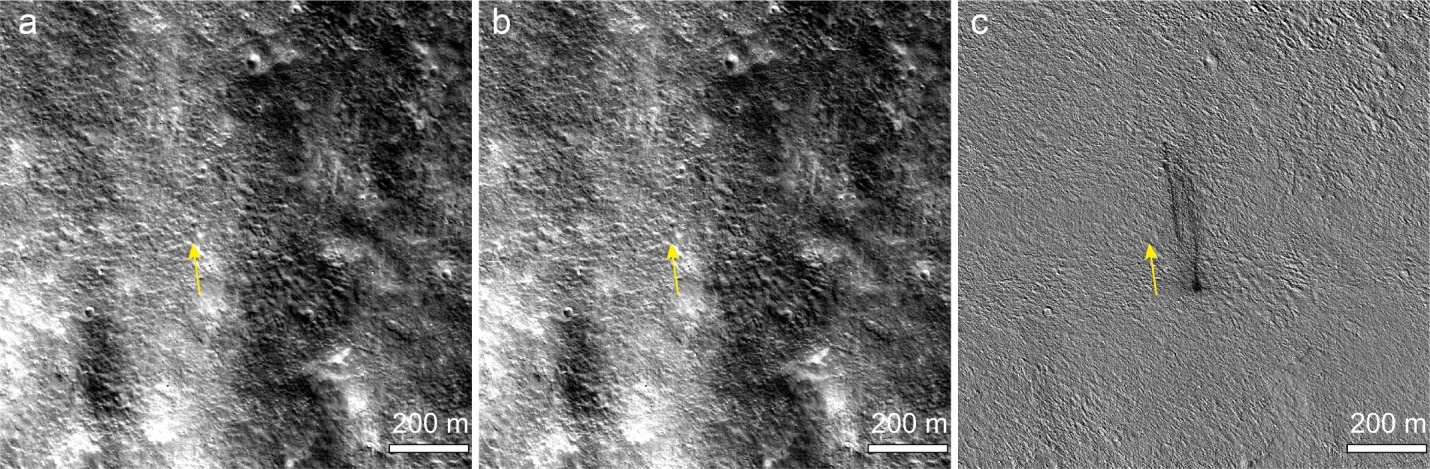


Supplementary Fig. S10. A new landslide that was likely triggered by a new impact on the southern crater wall of Timocharis. (a–c) Before, after and temporal ratio images, respectively. The coordinates of the new landslide are 26.221°N, 13.041°W. Yellow arrows point to downslope directions. The new impact is visible right in the initiation zone of the landslides, and it occurs as splotch caused by impact rays [8], while the topography of impact crater is not discernible due to its small size. IDs and addresses of data used in this figure are available at Table 3.


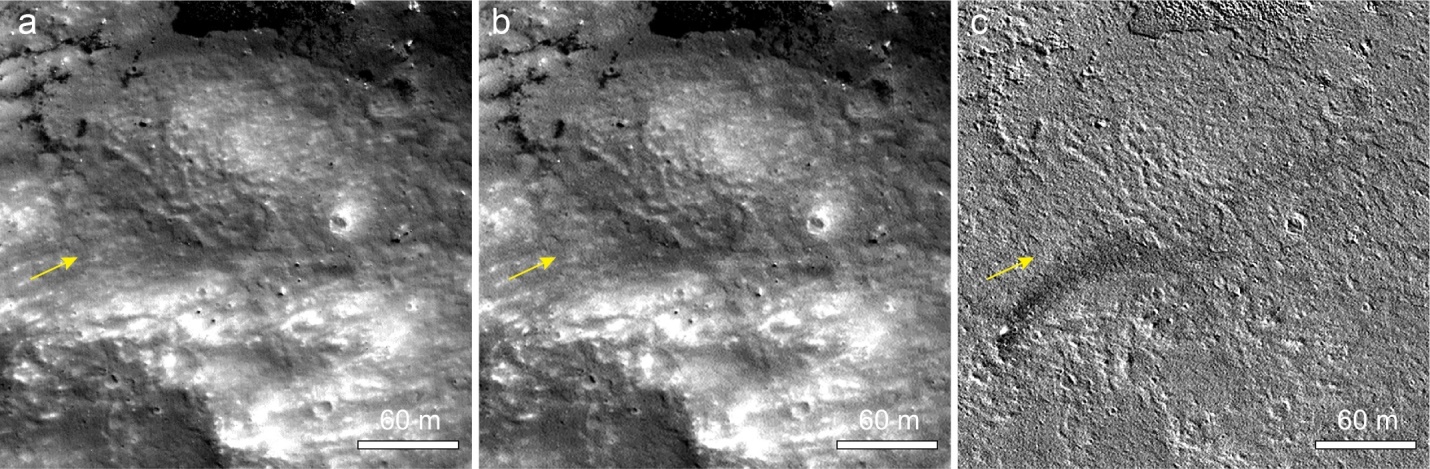


Supplementary Fig. S11. A new landslide that was likely triggered by a new impact on the western crater wall of Tycho. (a–c) Before, after and temporal ratio images, respectively. The coordinates of the new landslide are 43.346°S, 13.034°W. Yellow arrows point to downslope directions. The new impact is visible at the immediate heads of the landslides, and it occurs as a bright splotch caused by impact rays [8], while the topography of impact crater is not discernible due to the small size. IDs and addresses of data used in this figure are available at Table 3.


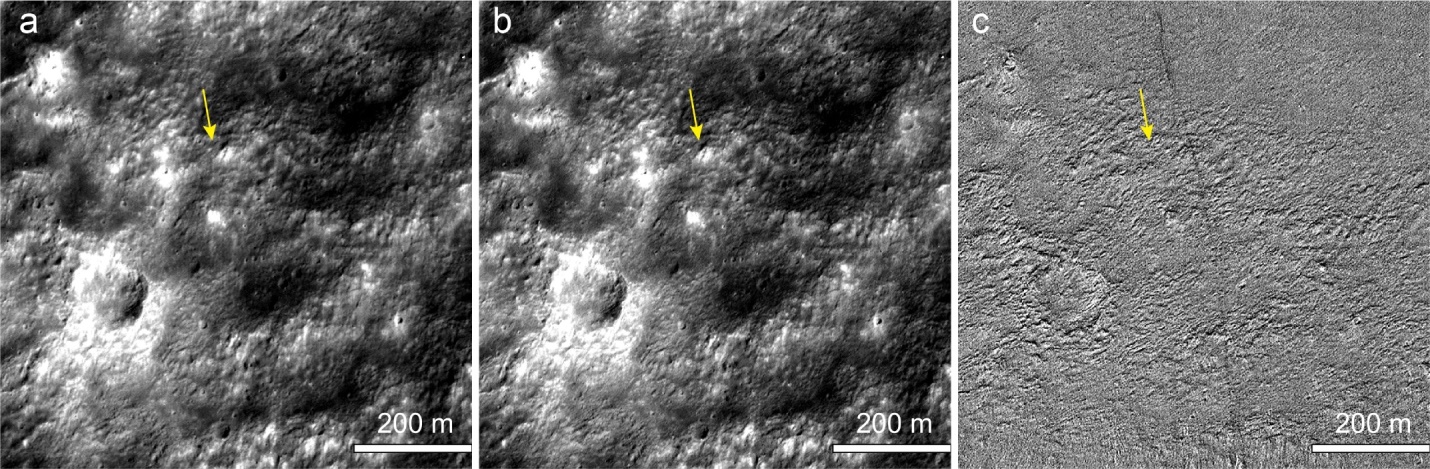


Supplementary Fig. S12. A new landslide that was likely triggered by endogenic seismic activity on the northern crater wall of Archimedes. (a–c) Before, after and temporal ratio images, respectively. The coordinates of the new landslide are 30.902°N, 4.460°W. Yellow arrows point to downslope directions. The new impact is visible right in the initiation zone of the landslides, and it occurs as splotch caused by impact rays [8], while the topography of impact crater is not discernible due to its small size. IDs and addresses of data used in this figure are available at Table 3.


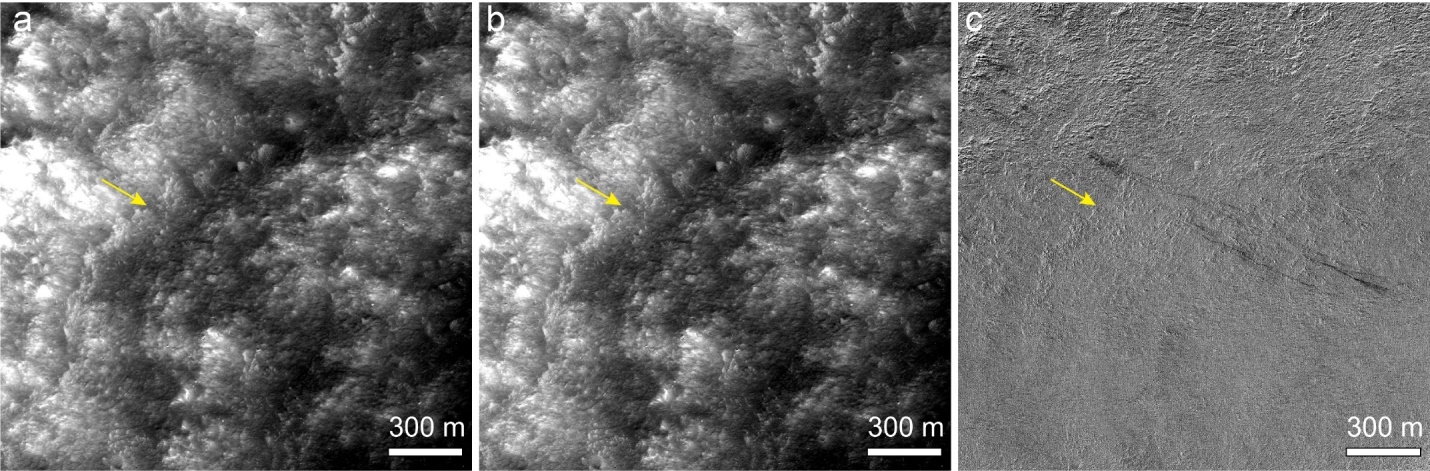


Supplementary Fig. S13. Two new landslides that were likely triggered by endogenic seismic activity on the northwestern crater wall of Aristillus. (a–c) Before, after and temporal ratio images, respectively. The central coordinates of this frame are 34.190°N, 0.336°E. Yellow arrows point to downslope directions. IDs and addresses of data used in this figure are available at Table 3.


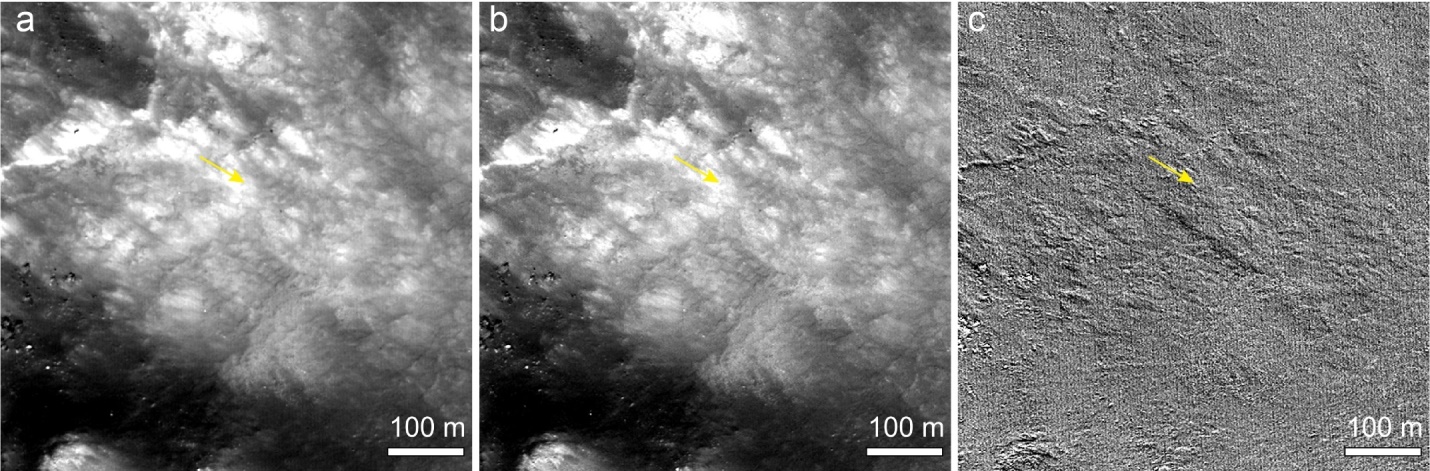


Supplementary Fig. S14. A new landslide that was likely triggered by endogenic seismic activity on the northwestern crater wall of Aristillus. (a–c) Before, after and temporal ratio images, respectively. The coordinates of the new landslide are 34.270°N, 0.360°E. Yellow arrows point to downslope directions. IDs and addresses of data used in this figure are available at Table 3.


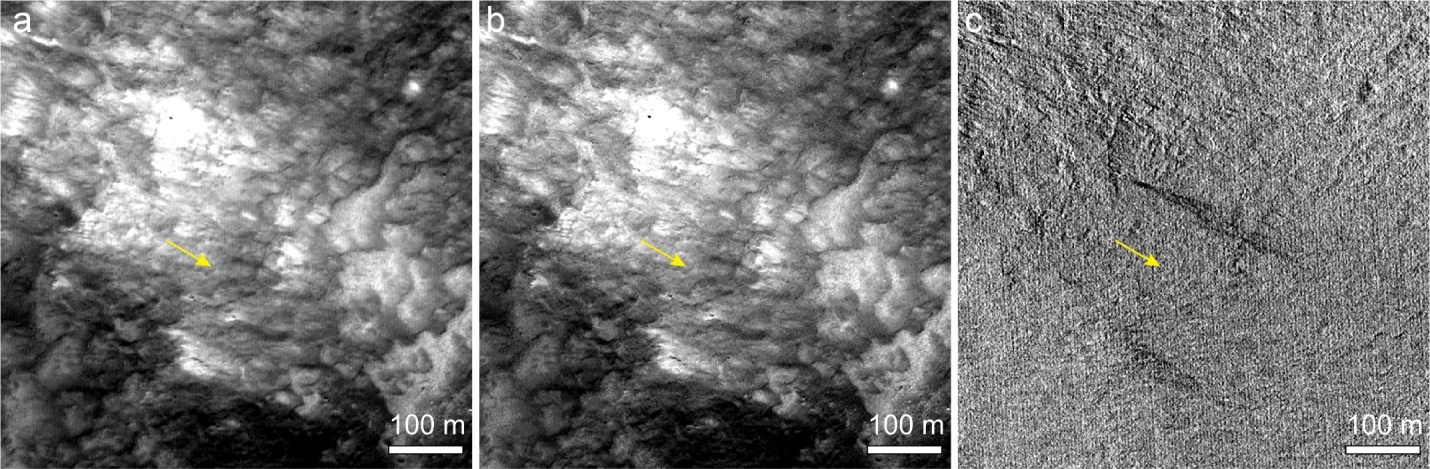


Supplementary Fig. S15. Two new landslides that were likely triggered by endogenic seismic activity on the northwestern crater wall of Aristillus. (a–c) Before, after and temporal ratio images, respectively. The central coordinates of this frame are 34.230°N, 0.390°E. Yellow arrows point to downslope directions. IDs and addresses of data used in this figure are available at Table 3.


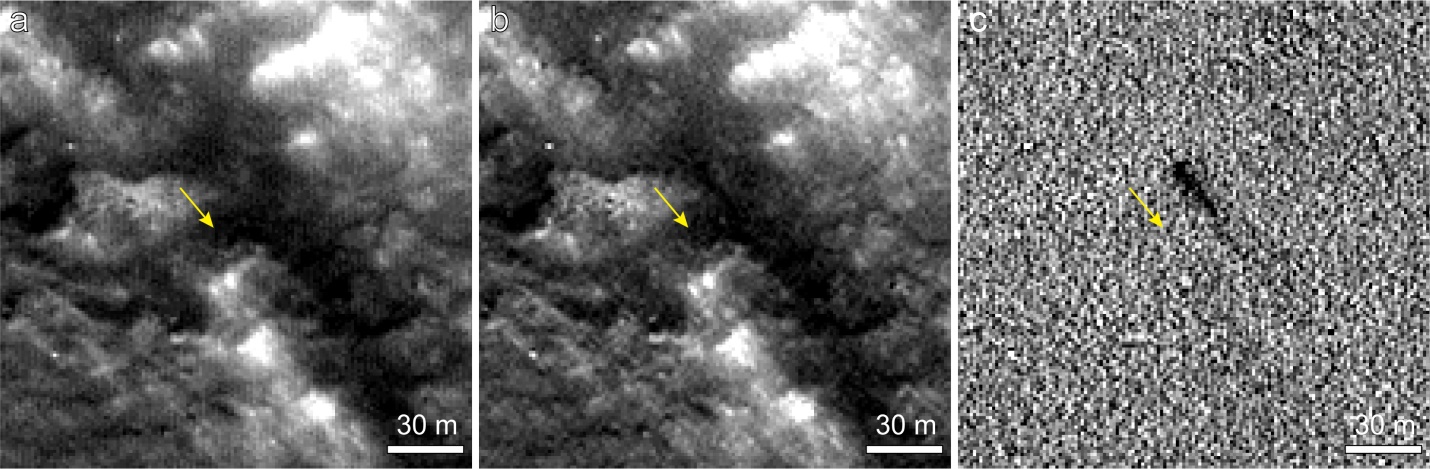


Supplementary Fig. S16. A new landslide that was likely triggered by endogenic seismic activity on the northwestern crater wall of Aristillus. (a–c) Before, after and temporal ratio images, respectively. The coordinates of the new landslide are 34.400°N, 0.577°E. Yellow arrows point to downslope directions. IDs and addresses of data used in this figure are available at Table 3.


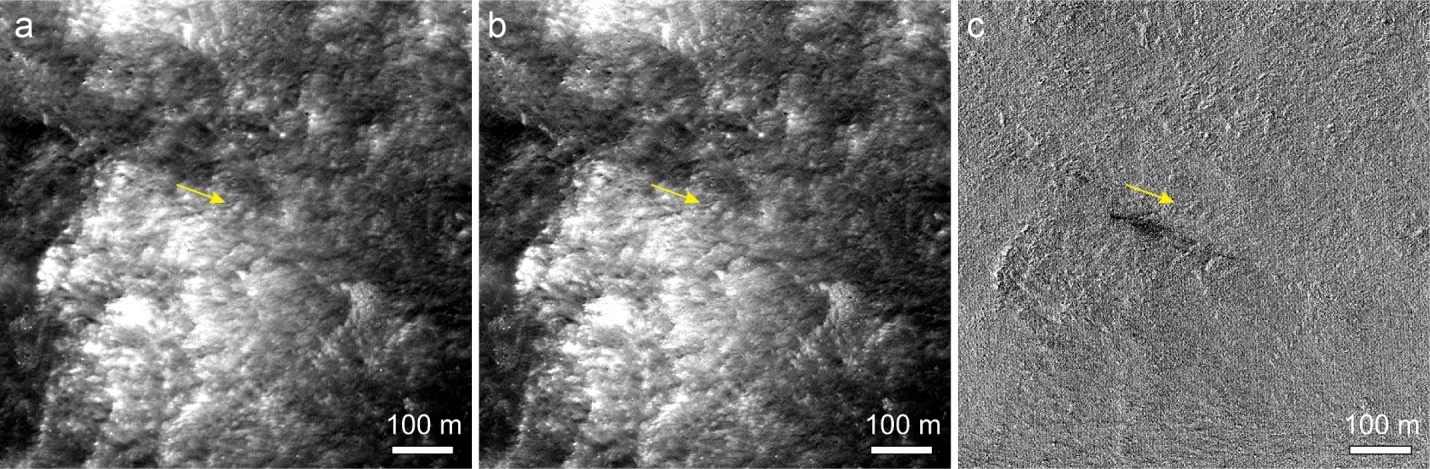


Supplementary Fig. S17. A new landslide that was likely triggered by endogenic seismic activity on the northwestern crater wall of Aristillus. (a–c) Before, after and temporal ratio images, respectively. The coordinates of the new landslide are 34.010°N, 0.330°E. Yellow arrows point to downslope directions. IDs and addresses of data used in this figure are available at Table 3.


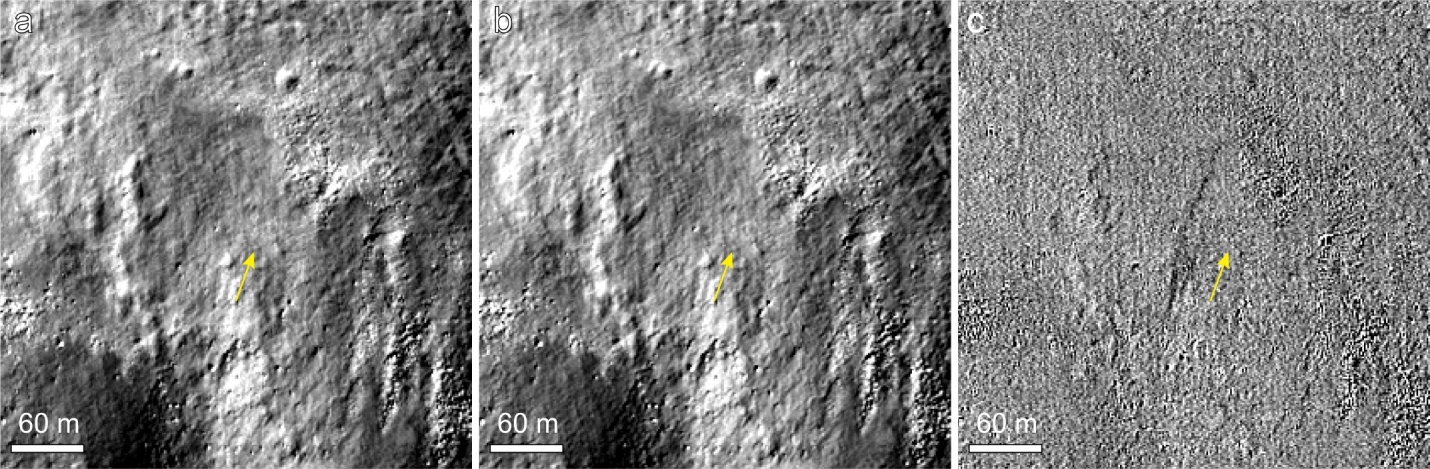


Supplementary Fig. S18. A new landslide that was likely triggered by endogenic seismic activity on the southern crater wall of Aristillus A. (a–c) Before, after and temporal ratio images, respectively. The coordinates of the new landslide are 33.595°N, 4.526°E. Yellow arrows point to downslope directions. IDs and addresses of data used in this figure are available at Table 3.


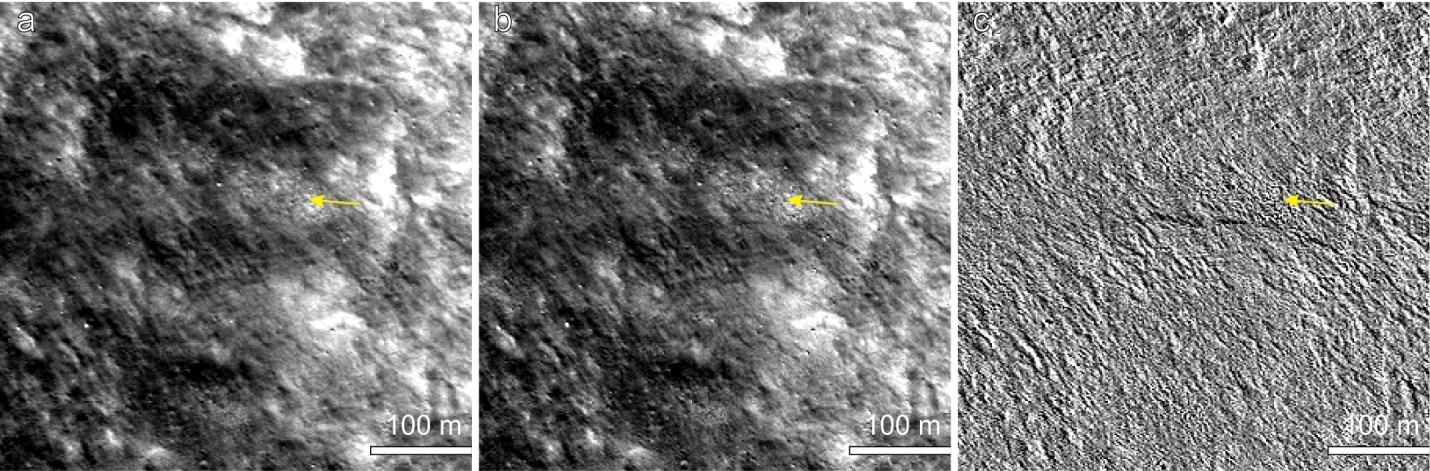


Supplementary Fig. S19. A new landslide that was likely triggered by endogenic seismic activity on the eastern crater wall of Aristillus B. (a–c) Before, after and temporal ratio images, respectively. The coordinates of the new landslide are 34.774°N, 1.816°W. Yellow arrows point to downslope directions. IDs and addresses of data used in this figure are available at Table 3.


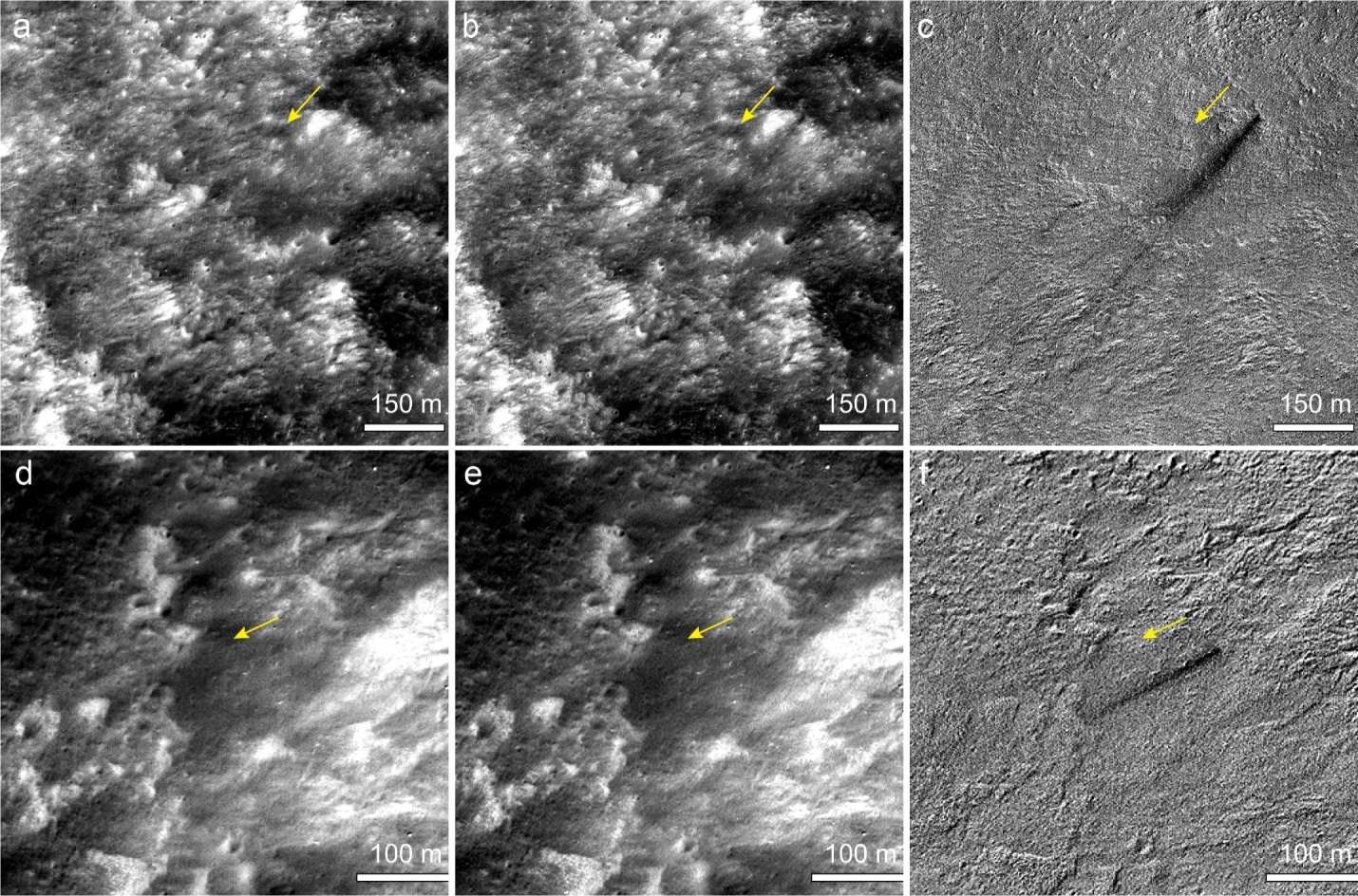


Supplementary Fig. S20. A new landslide that was likely triggered by endogenic seismic activity on the northeastern crater wall of Autolycus. (a–c) Before, after and temporal ratio images of the new landslide that are centered at 31.114°N, 1.962°E, respectively. (D–f) Before, after and temporal ratio images of the new landslide that are centered at 31.023°N, 2.027°E, respectively. Yellow arrows point to downslope directions. IDs and addresses of data used in this figure are available at Table 3.


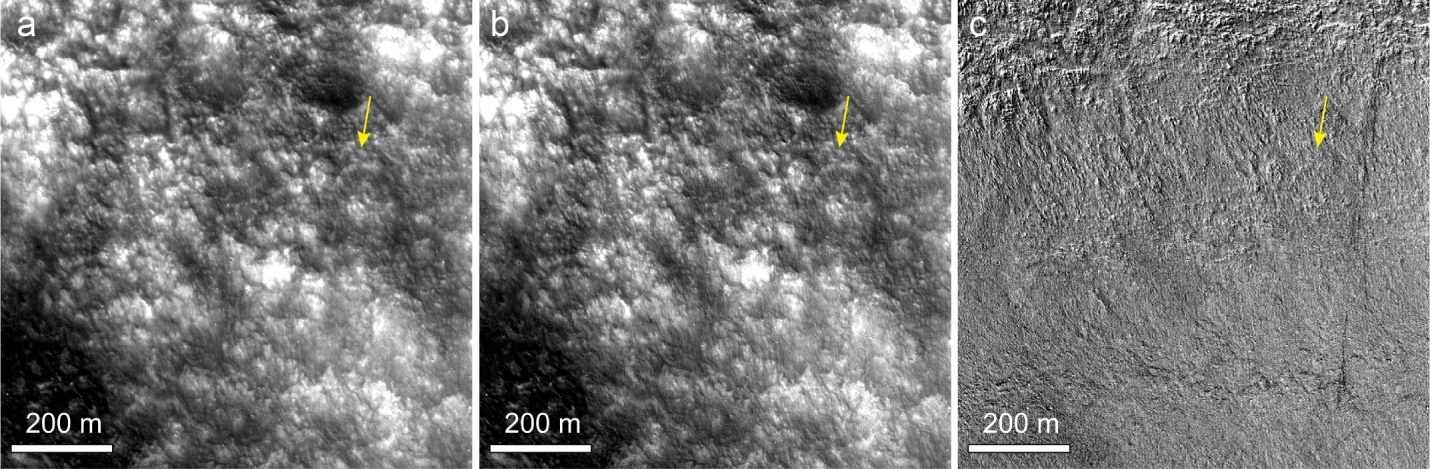


Supplementary Fig. S21. A new landslide that was likely triggered by endogenic seismic activity on the northern crater wall of Autolycus. (a–c) Before, after and temporal ratio images, respectively. The coordinates of the new landslide are 31.308°N, 1.360°E. Yellow arrows point to downslope directions. IDs and addresses of data used in this figure are available at Table 3.


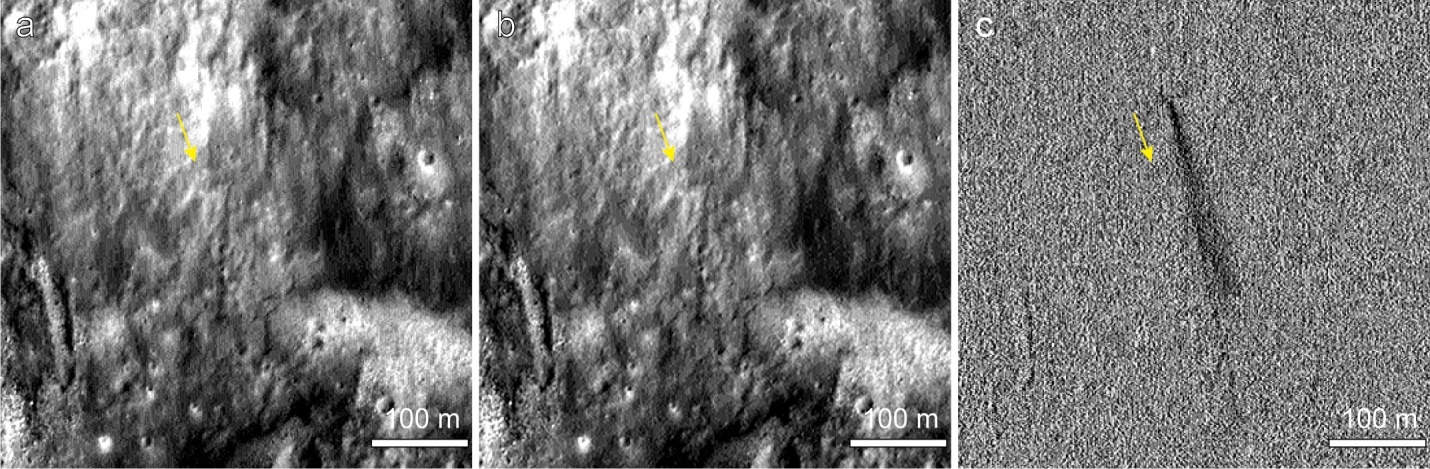


Supplementary Fig. S22. A new landslide that was likely triggered by endogenic seismic activity on the northern crater wall of Briggs B. (a–c) Before, after and temporal ratio images, respectively. The coordinates of the new landslide are 28.499°N, 70.992°W. Yellow arrows point to downslope directions. IDs and addresses of data used in this figure are available at Table 3.


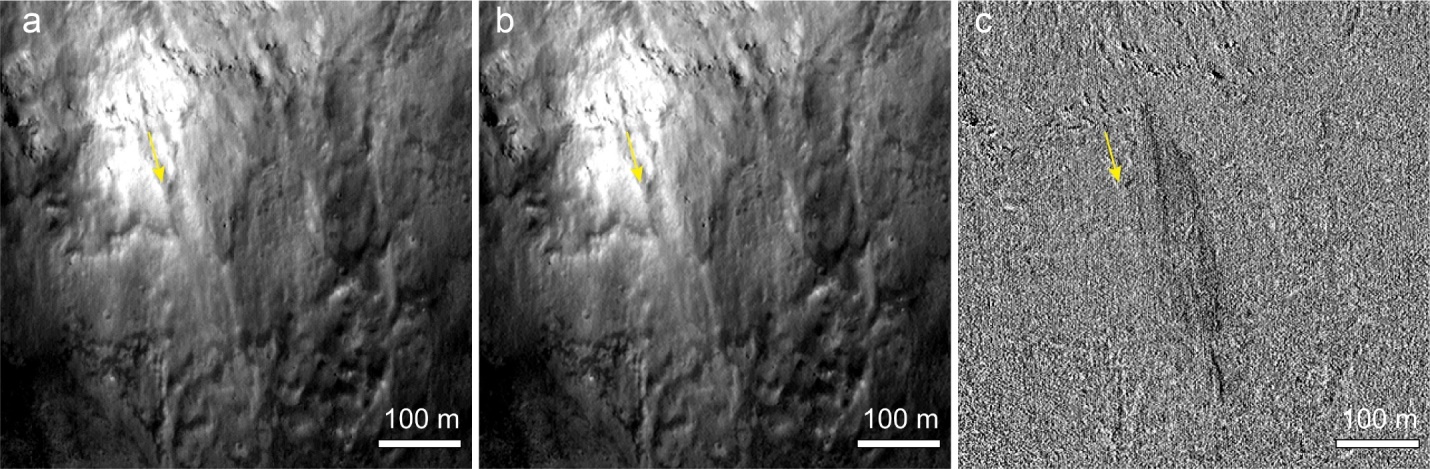


Supplementary Fig. S23. A new landslide that was likely triggered by endogenic seismic activity on the northern crater wall of Briggs B. (a–c) Before, after and temporal ratio images, respectively. The coordinates of the new landslide are 28.519°N, 70.973°W. Yellow arrows point to downslope directions. IDs and addresses of data used in this figure are available at Table 3.


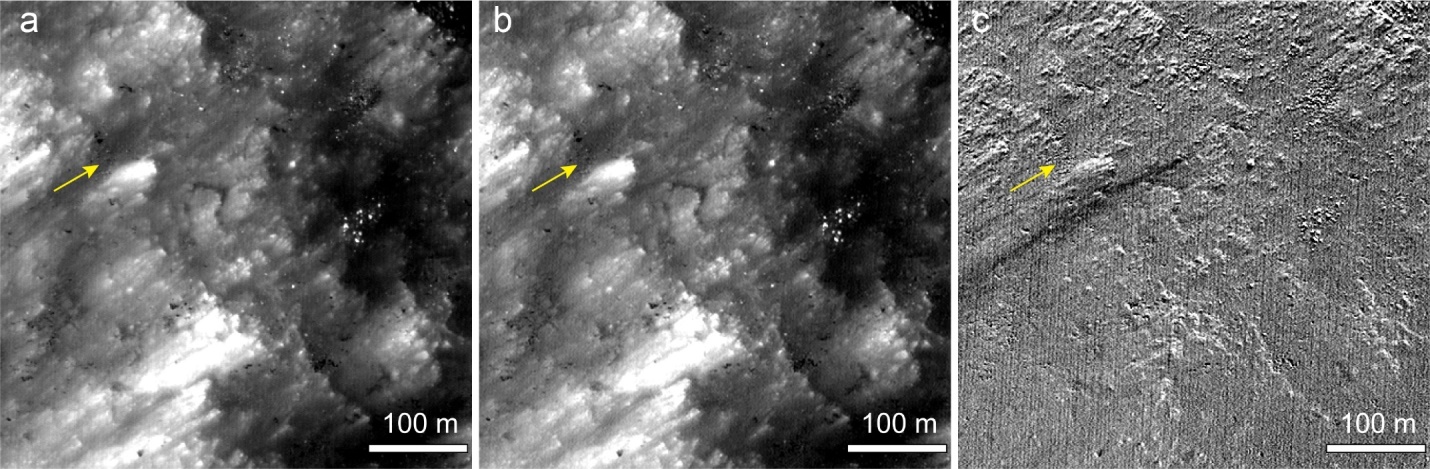


Supplementary Fig. S24. A new landslide that was likely triggered by endogenic seismic activity on the northeastern crater wall of Gambart A. (a–c) Before, after and temporal ratio images, respectively. The coordinates of the new landslide are 1.053°N, 18.617°W. Yellow arrows point to downslope directions. IDs and addresses of data used in this figure are available at Table 3.


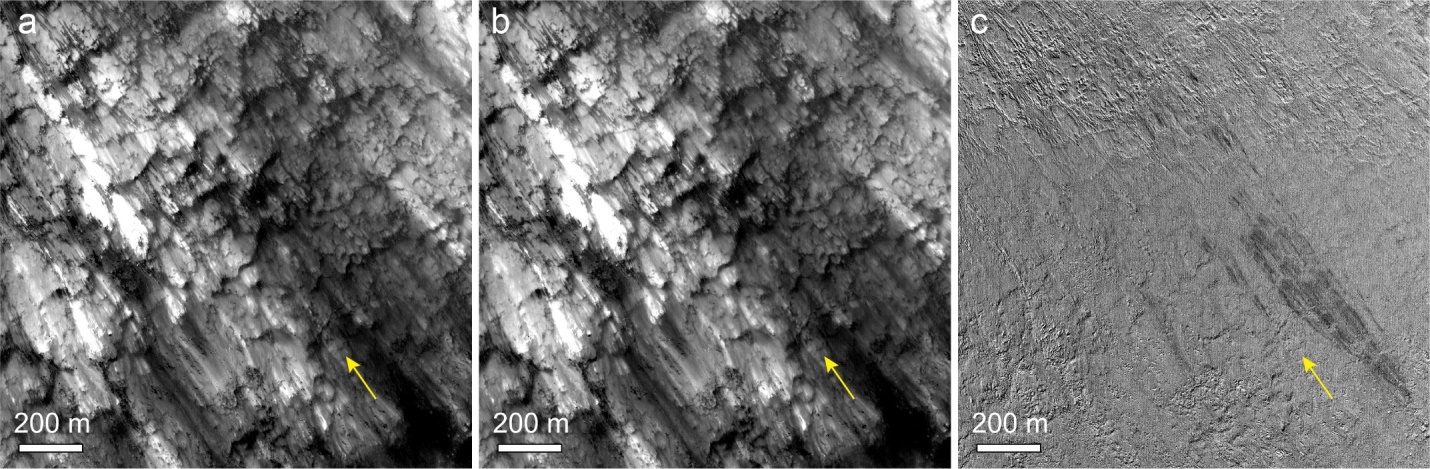


Supplementary Fig. S25. At least three new landslides that were likely triggered by endogenic seismic activity on the northern crater wall of Gambart A. (a–c) Before, after and temporal ratio images, respectively. The central coordinates of this new frame are 0.840°N, 18.680°W. Yellow arrows point to downslope directions. IDs and addresses of data used in this figure are available at Table 3.


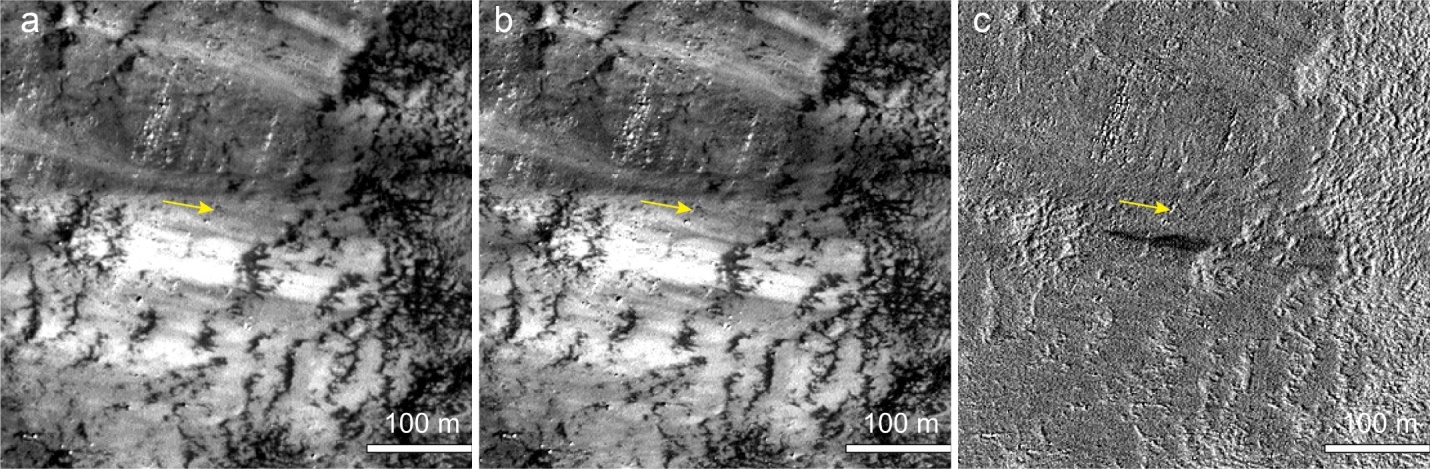


Supplementary Fig. S26. A new landslide that was likely triggered by endogenic seismic activity on the western crater wall of Heis. (a–c) Before, after and temporal ratio images, respectively. The coordinates of the new landslide are 32.547°N, 32.208°W. Yellow arrows point to downslope directions. IDs and addresses of data used in this figure are available at Table 3.


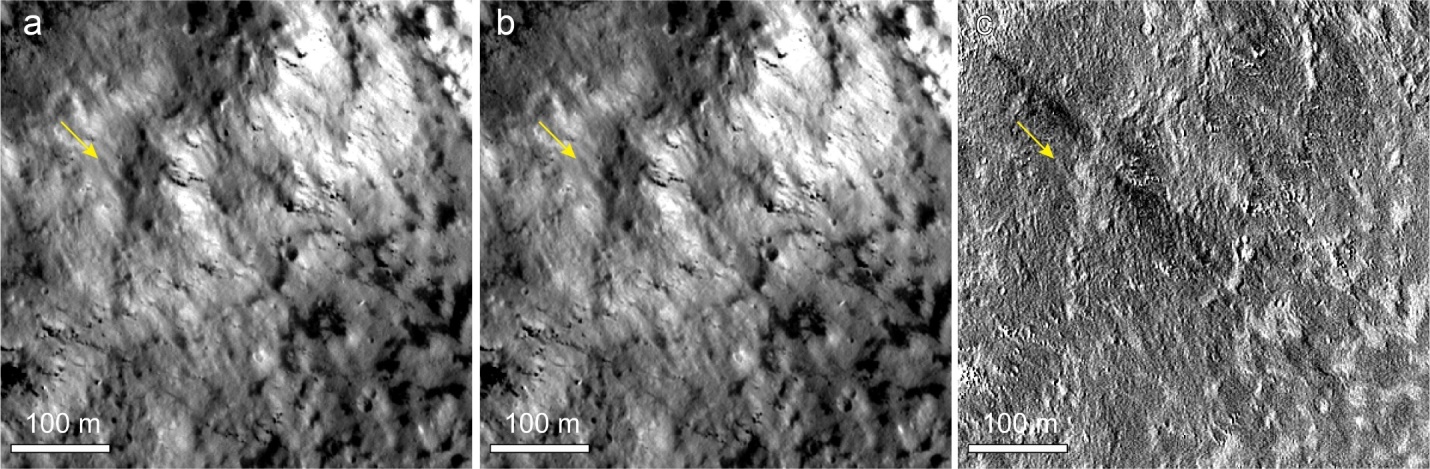


Supplementary Fig. S27. A new landslide that was likely triggered by endogenic seismic activity on the northwestern crater wall of Kepler. (a–c) Before, after and temporal ratio images, respectively. The coordinates of the new landslide are 8.506°N, 38.336°W. Yellow arrows point to downslope directions. IDs and addresses of data used in this figure are available at Table 3.


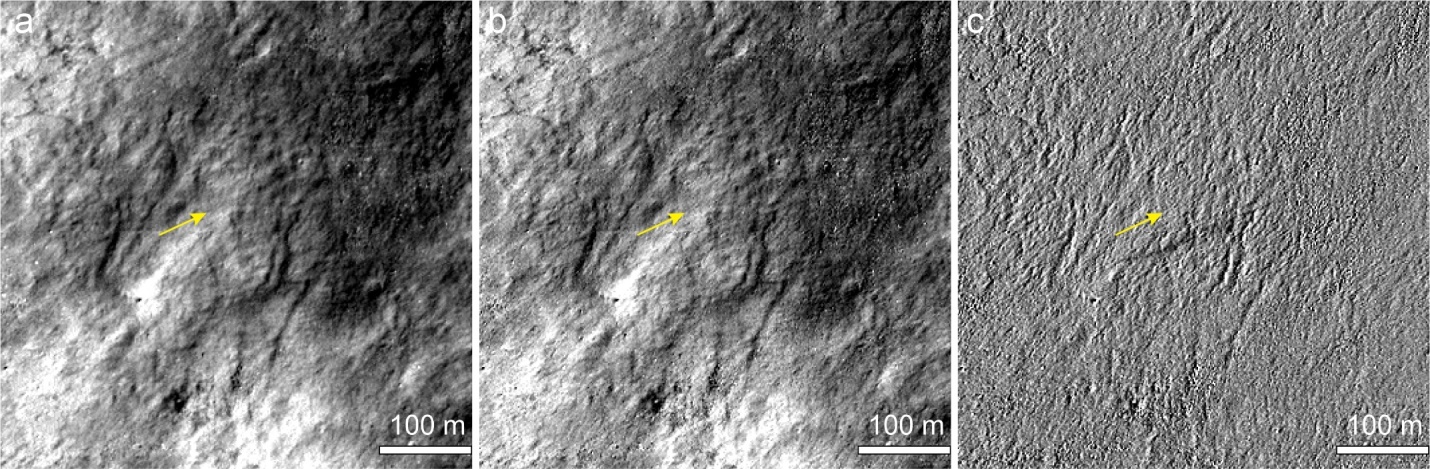


Supplementary Fig. S28. A new landslide that was likely triggered by endogenic seismic activity on the western crater wall of Kirch. (a–c) Before, after and temporal ratio images, respectively. The coordinates of the new landslide are 39.220°N, 5.804°W. Yellow arrows point to downslope directions. IDs and addresses of data used in this figure are available at Table 3.


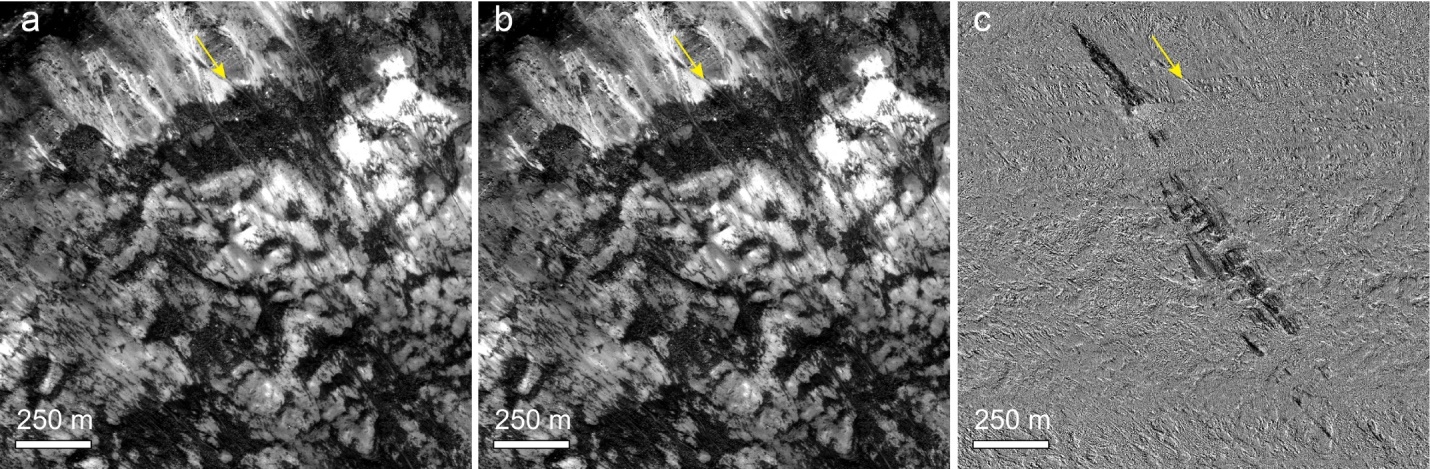


Supplementary Fig. S29. A new landslide that was likely triggered by endogenic seismic activity on the northwestern crater wall of Laplace A. (a–c) Before, after and temporal ratio images, respectively. The coordinates of the new landslide are 43.834°N, 27.033°W. Yellow arrows point to downslope directions. IDs and addresses of data used in this figure are available at Table 3.


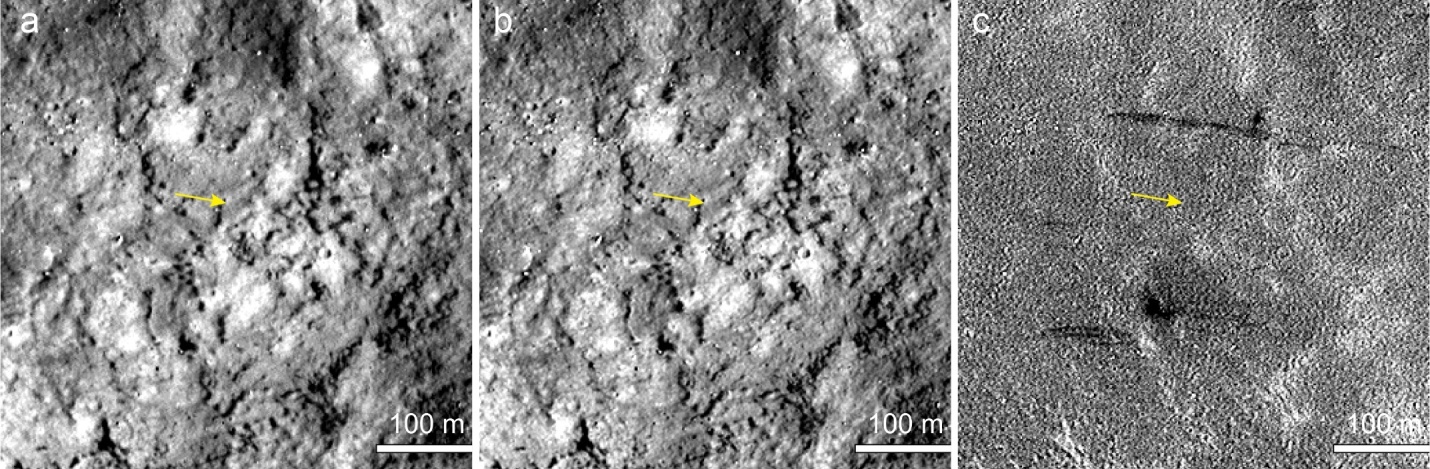


Supplementary Fig. S30. Several new landslides and new impacts on the western crater wall of Naumann. (a–c) Before, after and temporal ratio images, respectively. The coordinates of the new landslide are 35.390°N, 62.180°W. The new impact occurs as a splotch caused by impact rays [8], while the topography of impact crater is not discernible due to its small size. Yellow arrows point to downslope directions. IDs and addresses of data used in this figure are available at Table 3.


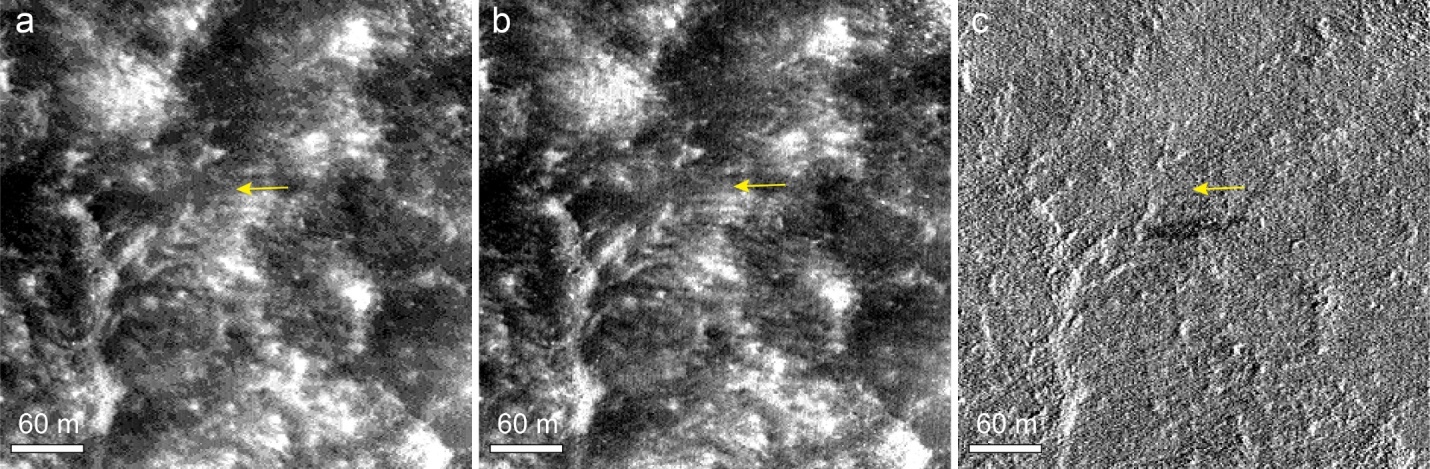


Supplementary Fig. S31. A new landslide that was likely triggered by endogenic seismic activity on the eastern crater wall of Pytheas. (a–c) Before, after and temporal ratio images, respectively. The coordinates of the new landslide are 20.630°N, 20.344°W. Yellow arrows point to downslope directions. IDs and addresses of data used in this figure are available at Table 3.


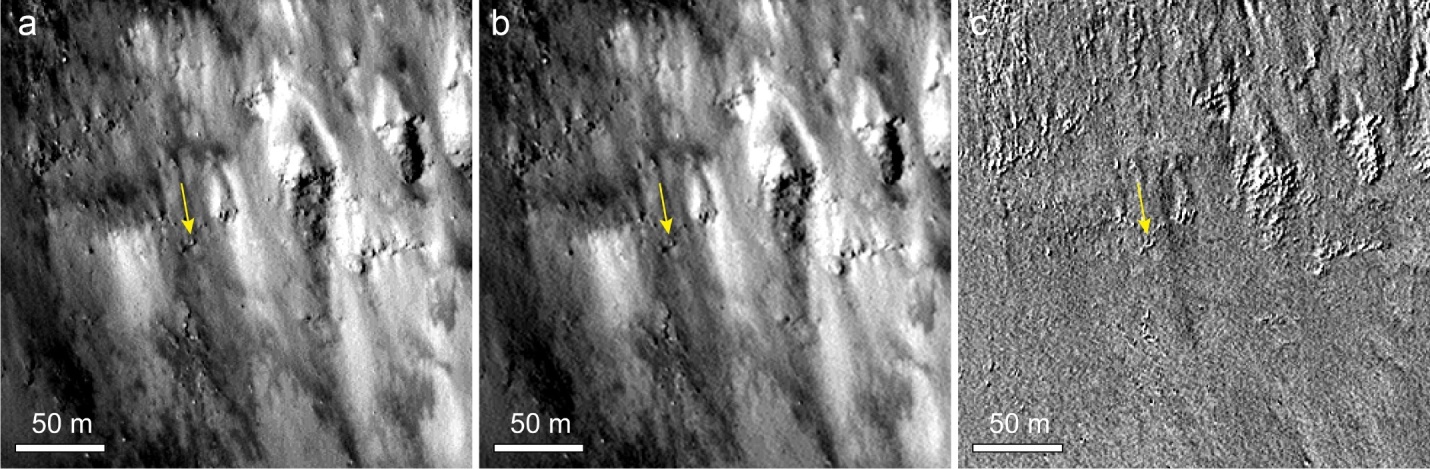


Supplementary Fig. S32. A new landslide that was likely triggered by endogenic seismic activity on the northern crater wall of Reiner. (a–c) Before, after and temporal ratio images, respectively. The coordinates of the new landslide are 7.354°N, 55.040°W. Yellow arrows point to downslope directions. IDs and addresses of data used in this figure are available at Table 3.


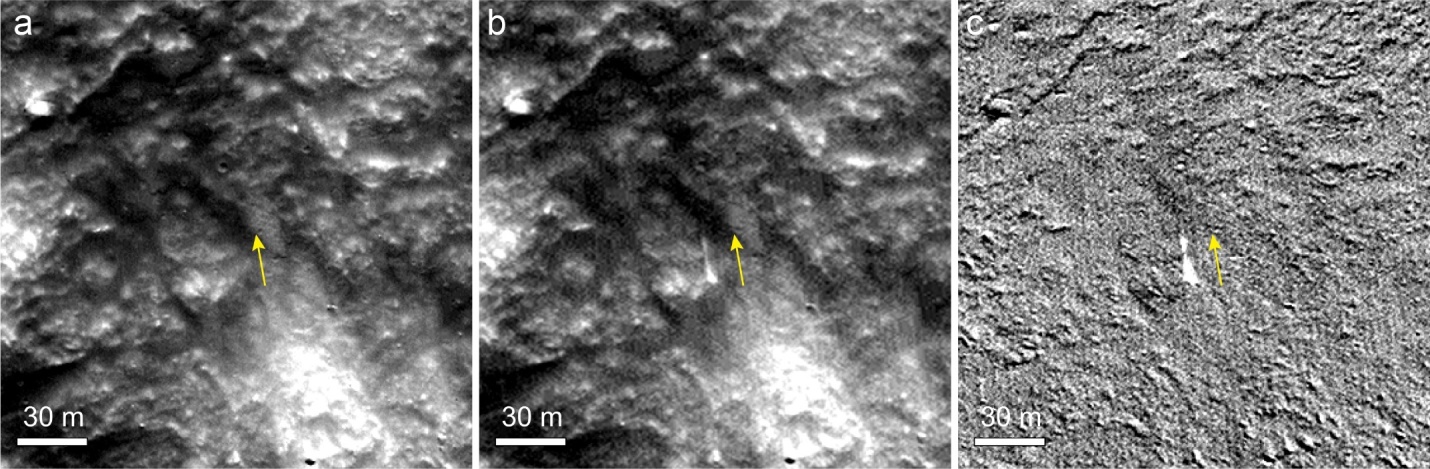


Supplementary Fig. S33. A new landslide that was likely triggered by endogenic seismic activity on the southern crater wall of Ryder. (a–c) Before, after and temporal ratio images, respectively. The coordinates of the new landslide are 44.043°S, 143.514°E. Yellow arrows point to downslope directions. IDs and addresses of data used in this figure are available at Table 3.


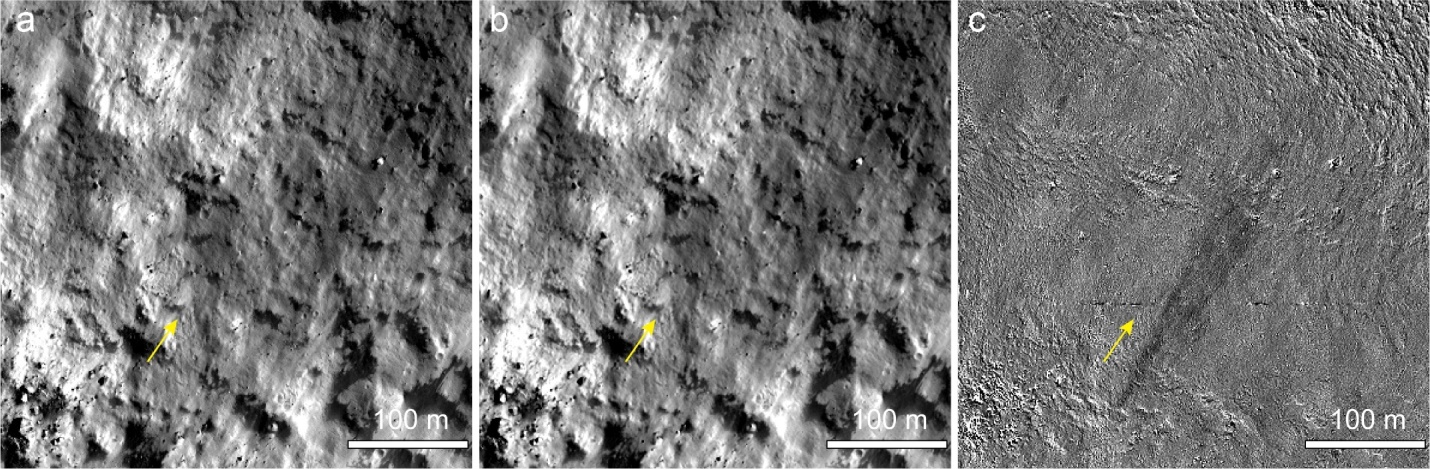


Supplementary Fig. S34. A new landslide that was likely triggered by endogenic seismic activity on the southwestern crater wall of Tycho. (a–c) Before, after and temporal ratio images, respectively. The coordinates of the new landslide are 44.123°S, 12.746°W. Yellow arrows point to downslope directions. IDs and addresses of data used in this figure are available at Table 3.


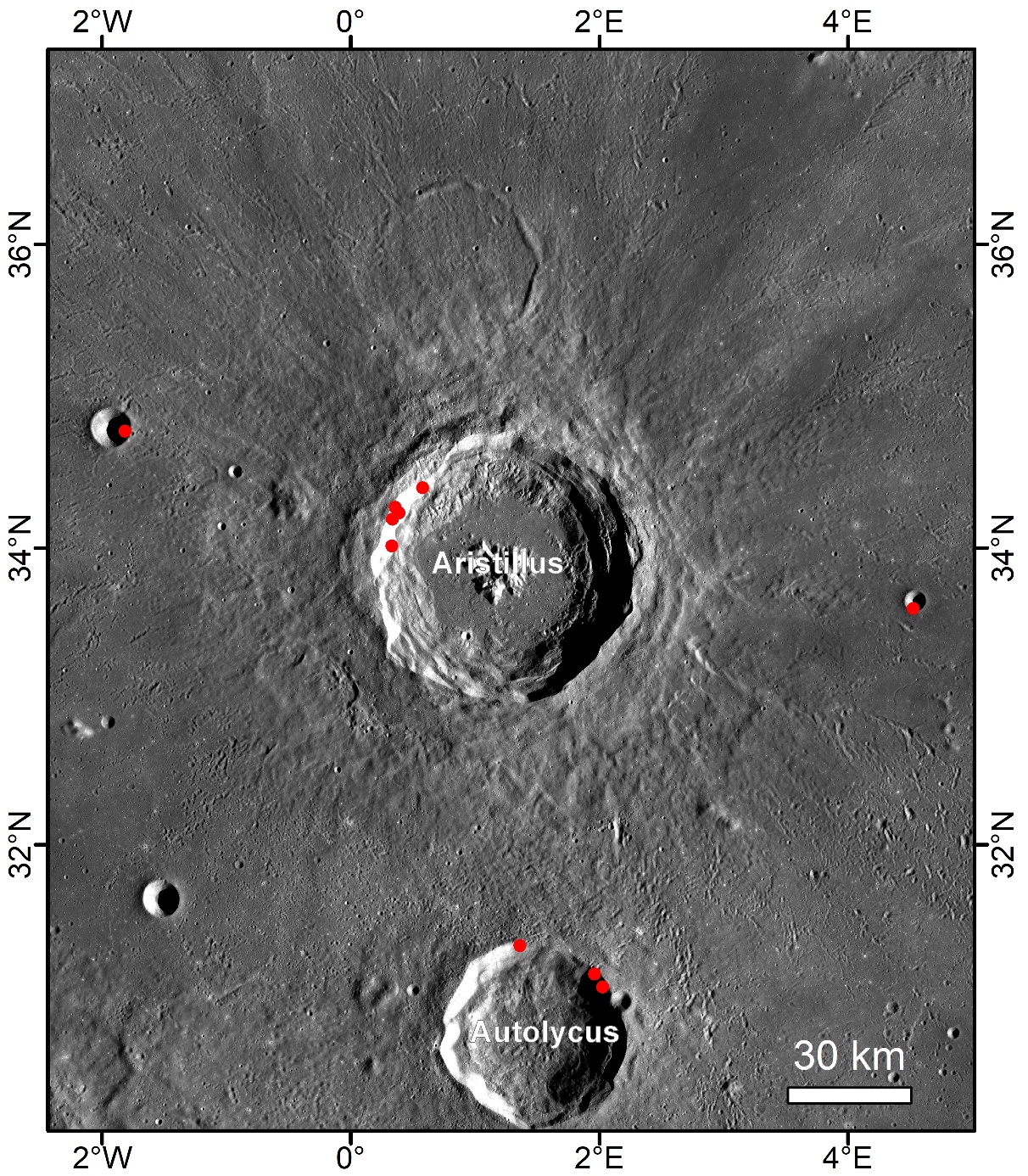


Supplementary Fig. S35. Clustered distribution of landslides triggered by endogenic activity on the western crater wall of Aristillus and northern crater wall of Autolycus. Red dots are recognized landslides triggered by endogenic seismic activity. IDs and addresses of data used in this figure are available at Table 3.


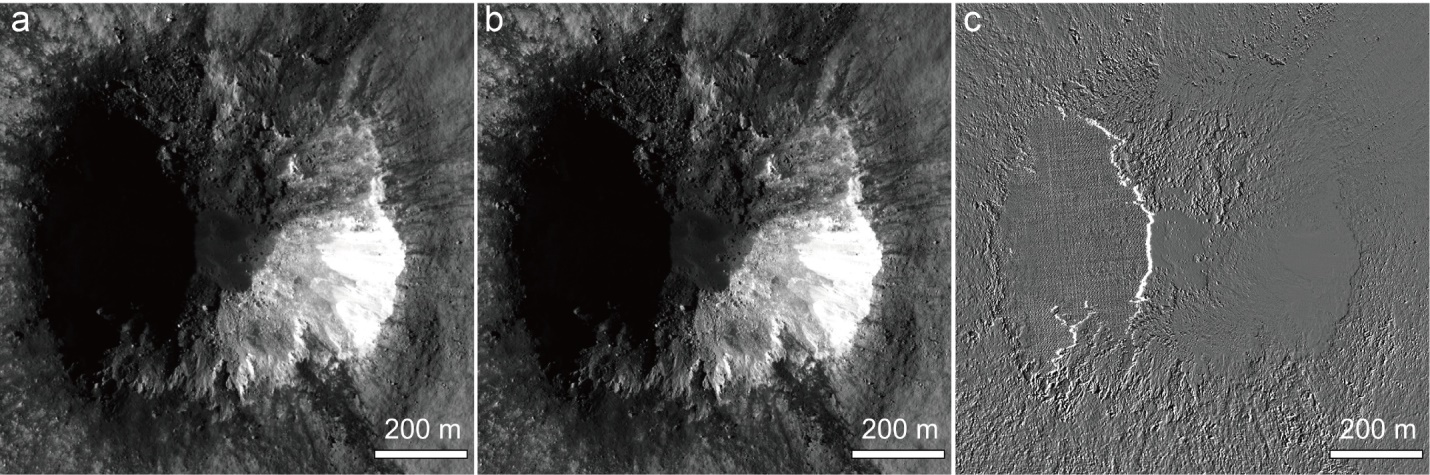


Supplementary Fig. S36. New landslides are not observed on the crater wall of Bandfield. (a–c) Before, after and temporal ratio images, respectively. Bandfield is a cold spot crater [25]. The central coordinates of this cold spot crater are 5.40°S, 90.77°E. IDs and addresses of data used in this figure are available at Table 3.


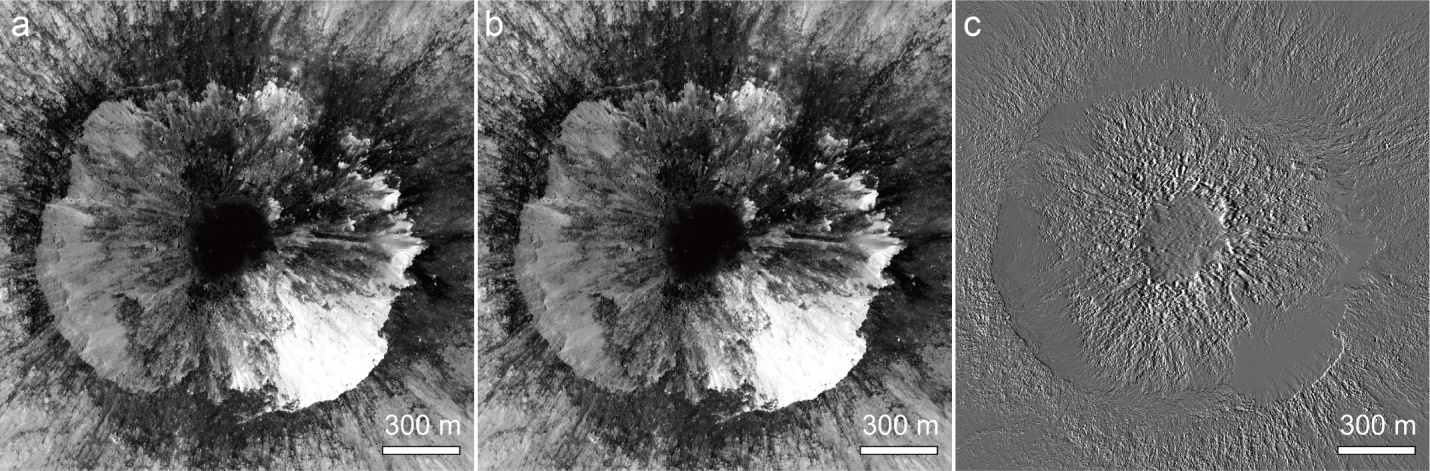


Supplementary Fig. S37. New landslides are not observed on the crater wall of Chaplygin B. (a–c) Before, after and temporal ratio images, respectively. Chaplygin B is a cold spot crater [25]. The coordinates of this cold spot crater are 4.08°S, 151.69°E. IDs and addresses of data used in this figure are available at Table 3.


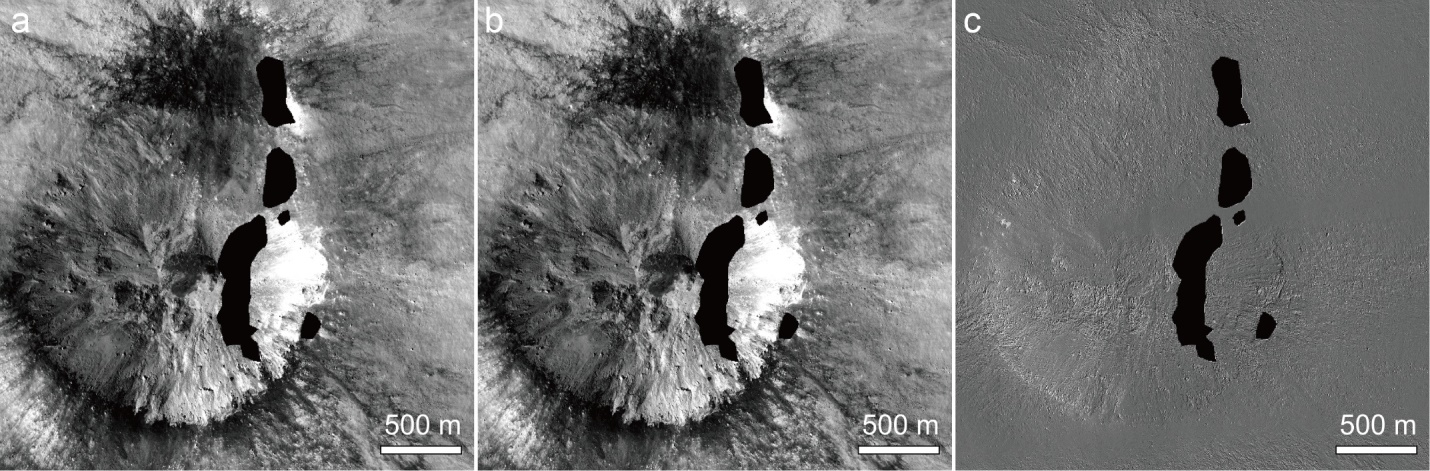


Supplementary Fig. S38. New landslides are not observed on the wall of an unnamed crater. (a–c) Before, after and temporal ratio images, respectively. This crater is a cold spot crater [25]. The coordinates of this cold spot crater are 18.688°N, 121.311°E. IDs and addresses of data used in this figure are available at Table 3.


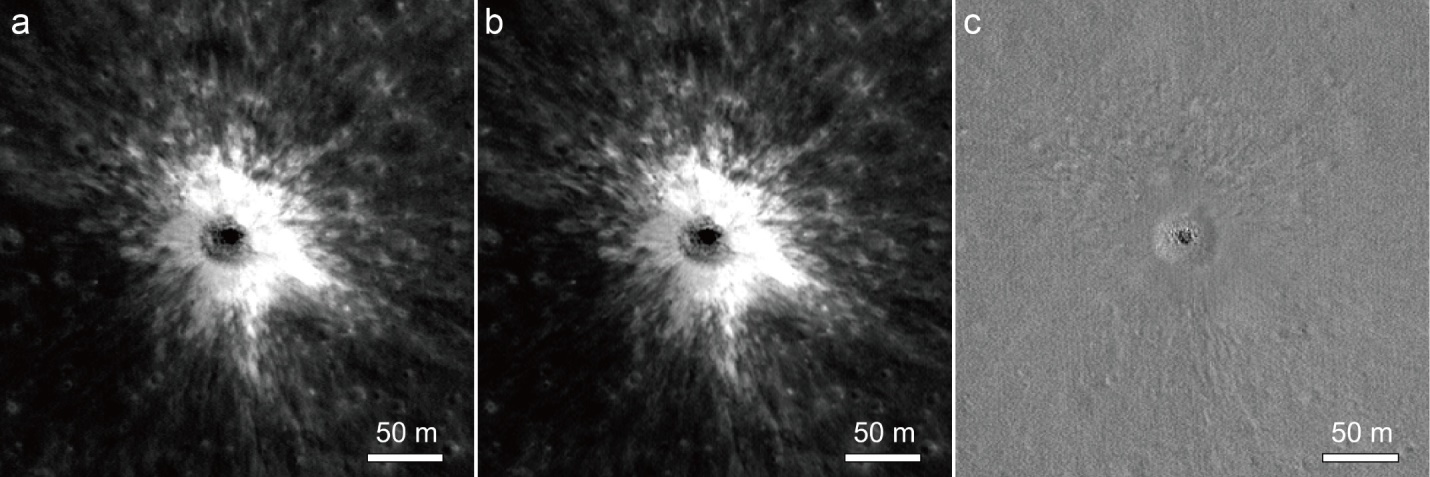


Supplementary Fig. S39. New landslides are not observed on the crater wall of New Crater 3. (a–c) Before, after and temporal ratio images, respectively. This crater was formed in the past 15 years [23]. The coordinates of this crater are 14.394°N, 28.271°W. IDs and addresses of data used in this figure are available at Table 3.


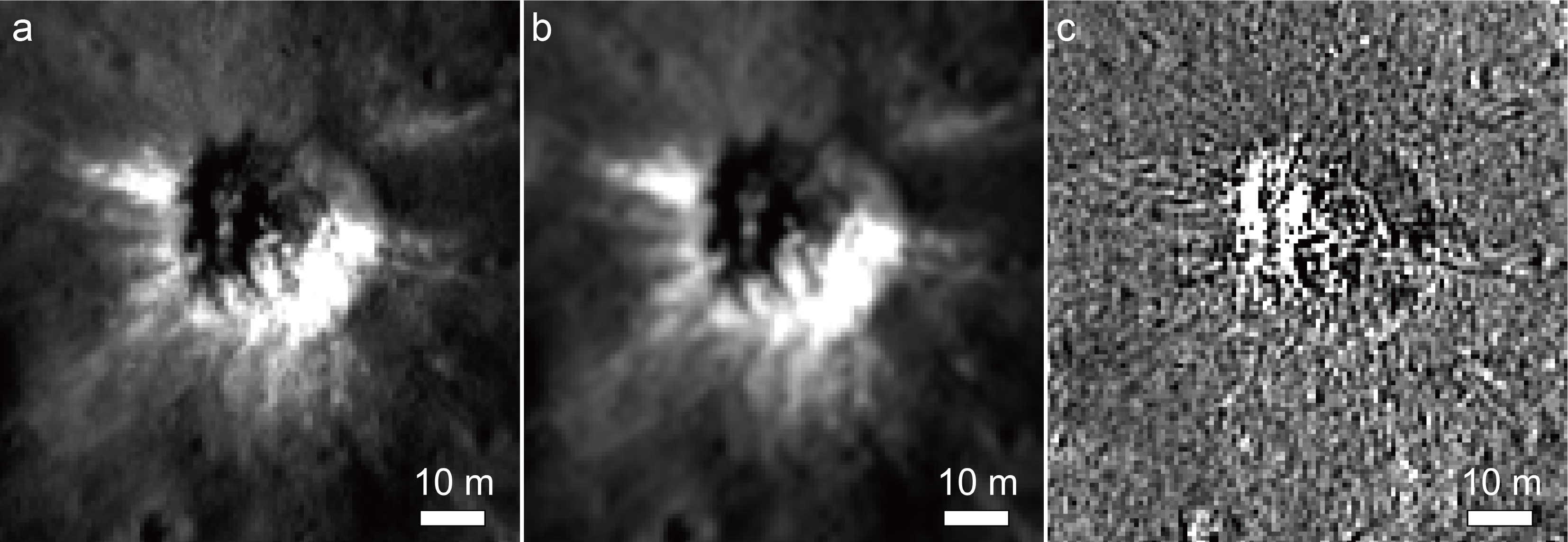


Supplementary Fig. S40. New landslides are not observed on the crater wall of New Crater 4. (a–c) Before, after and temporal ratio images, respectively. This crater was formed in the past 15 years [23]. The coordinates of this crater are 16.934°S, 85.649°W. IDs and addresses of data used in this figure are available at Table 3.


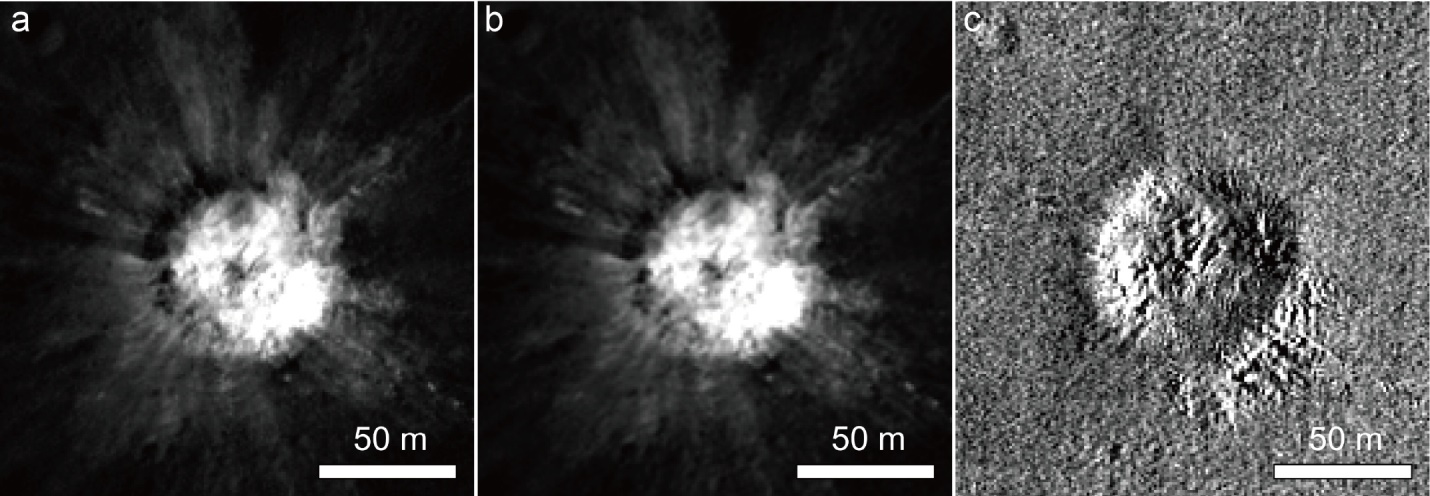


Supplementary Fig. S41. New landslides are not observed on the crater wall of New Crater 7. (a–c) Before, after and temporal ratio images, respectively. This crater was formed in the past 15 years [23]. The coordinates of this crater are 22.352°S, 39.73°W. IDs and addresses of data used in this figure are available at Table 3.


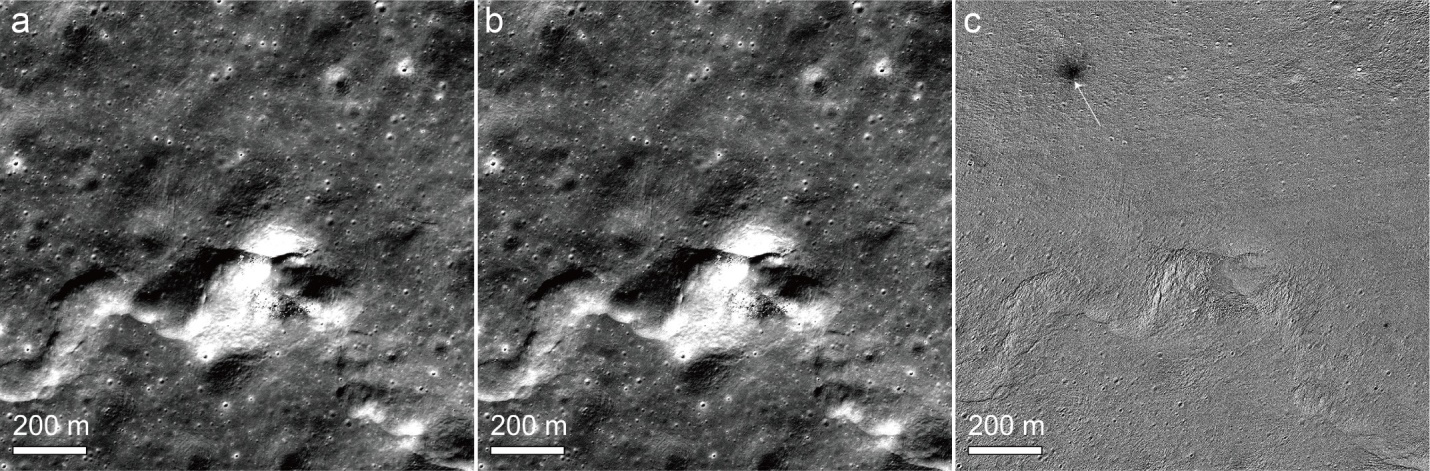


Supplementary Fig. S42. New landslides are not observed along a pristine wrinkle ridge located at 45.974°N, 4.751°W, but a new impact is visible to the north (white arrow). (a–c) Before, after and temporal ratio images, respectively. The new impact occurs as a splotch caused by impact rays [8], while the topography of impact crater is not discernible due to its small size. IDs and addresses of data used in this figure are available at Table 3.


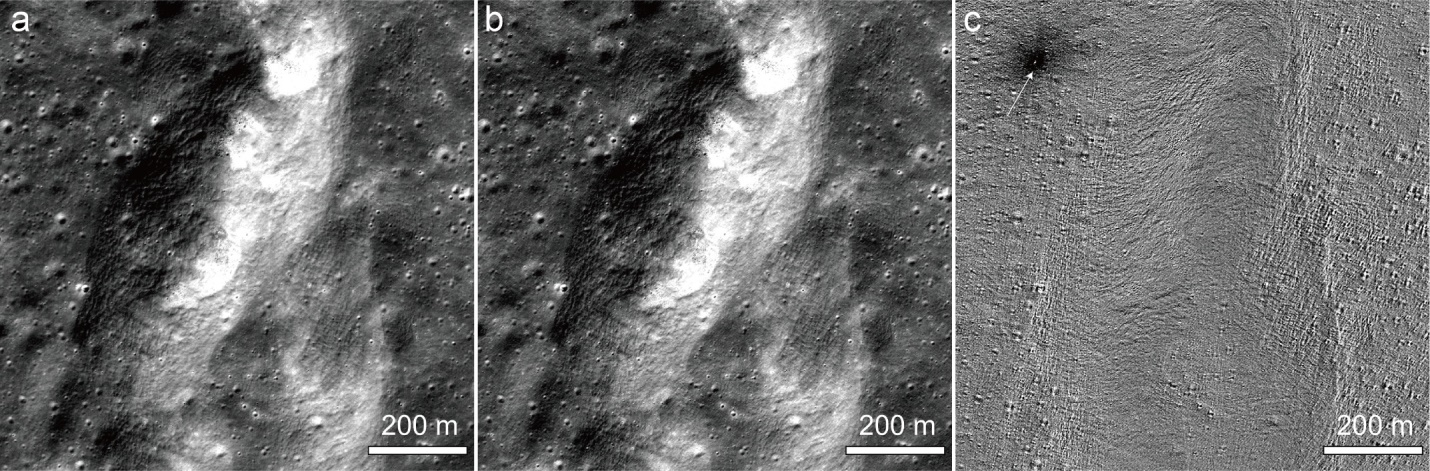


Supplementary Fig. S43. New landslides are not observed along a pristine wrinkle ridge located at 46.542°N, 4.841°W, but a new impact is visible to the northwest (white arrow). (a–c) Before, after and temporal ratio images, respectively. The new impact occurs as a splotch caused by impact rays [8], while the topography of impact crater is not discernible due to its small size. IDs and addresses of data used in this figure are available at Table 3.


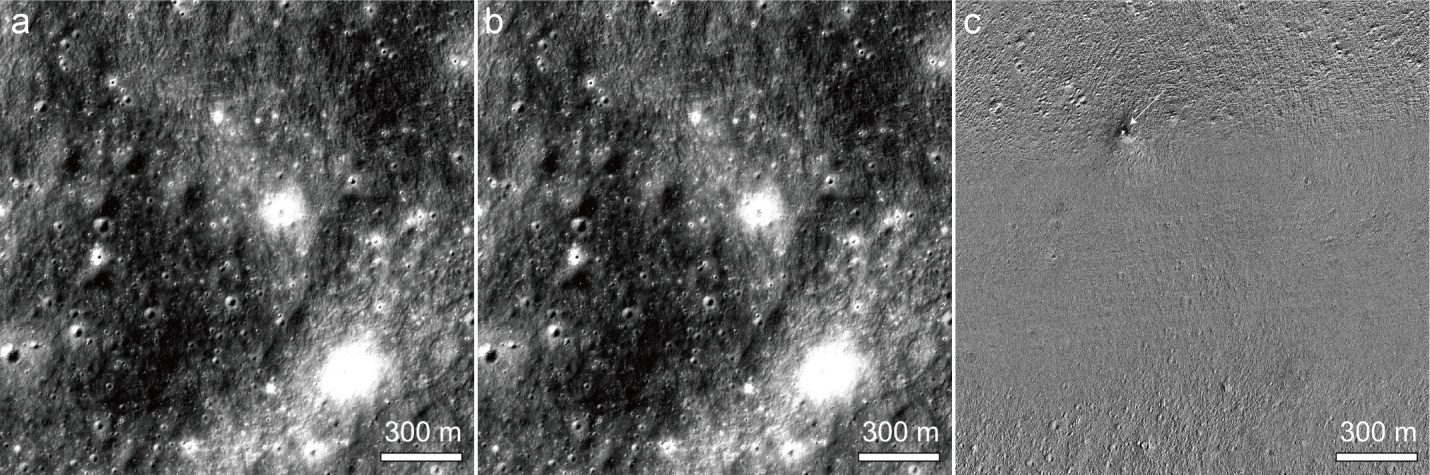


Supplementary Fig. S44. New landslides are not observed in a possible epicenter of strong shallow moonquakes recorded by Apollo [29], but a new impact is evident (white arrow). (a–c) Before, after and temporal ratio images, respectively. This area is for the interpreted epicenter 1 in early work [29]. The central coordinates of this area are 28.835°N, 97.851°W. The new impact occurs as a splotch caused by impact rays [8], while the topography of impact crater is not discernible due to its small size. IDs and addresses of data used in this figure are available at Table 3.


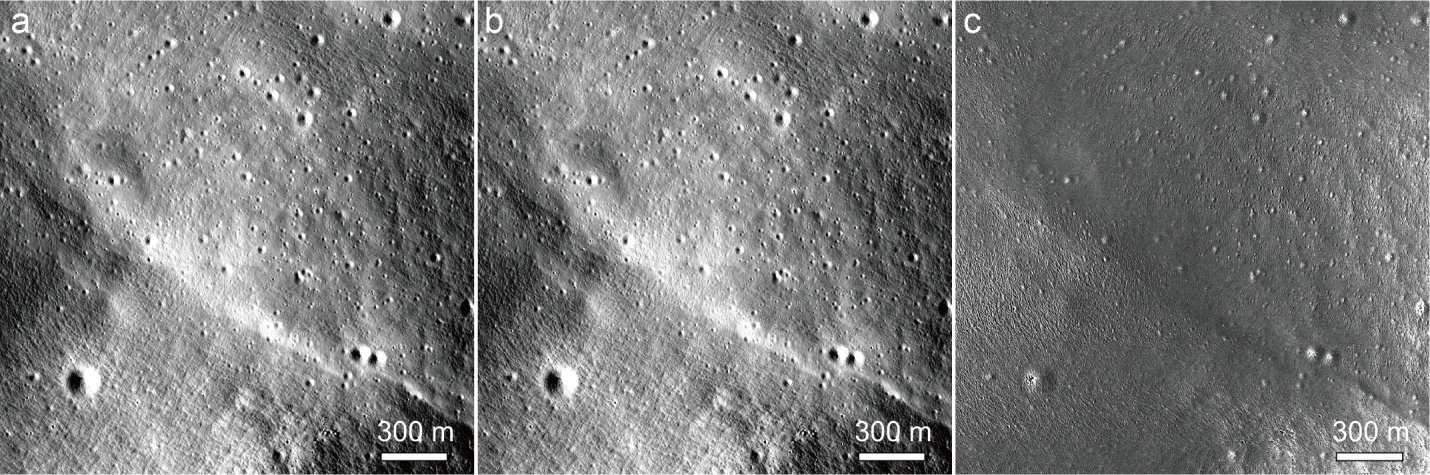


Supplementary Fig. S45. New landslides are not observed along a lobate scarp that was interpreted to be a possible epicenter of strong shallow moonquakes recorded by Apollo [29]. (a–c) Before, after and temporal ratio images, respectively. This area is for the interpreted epicenter 2 in early work [29]. The central coordinates of this area are 28.308°N, 98.113°W. IDs and addresses of data used in this figure are available at Table 3.


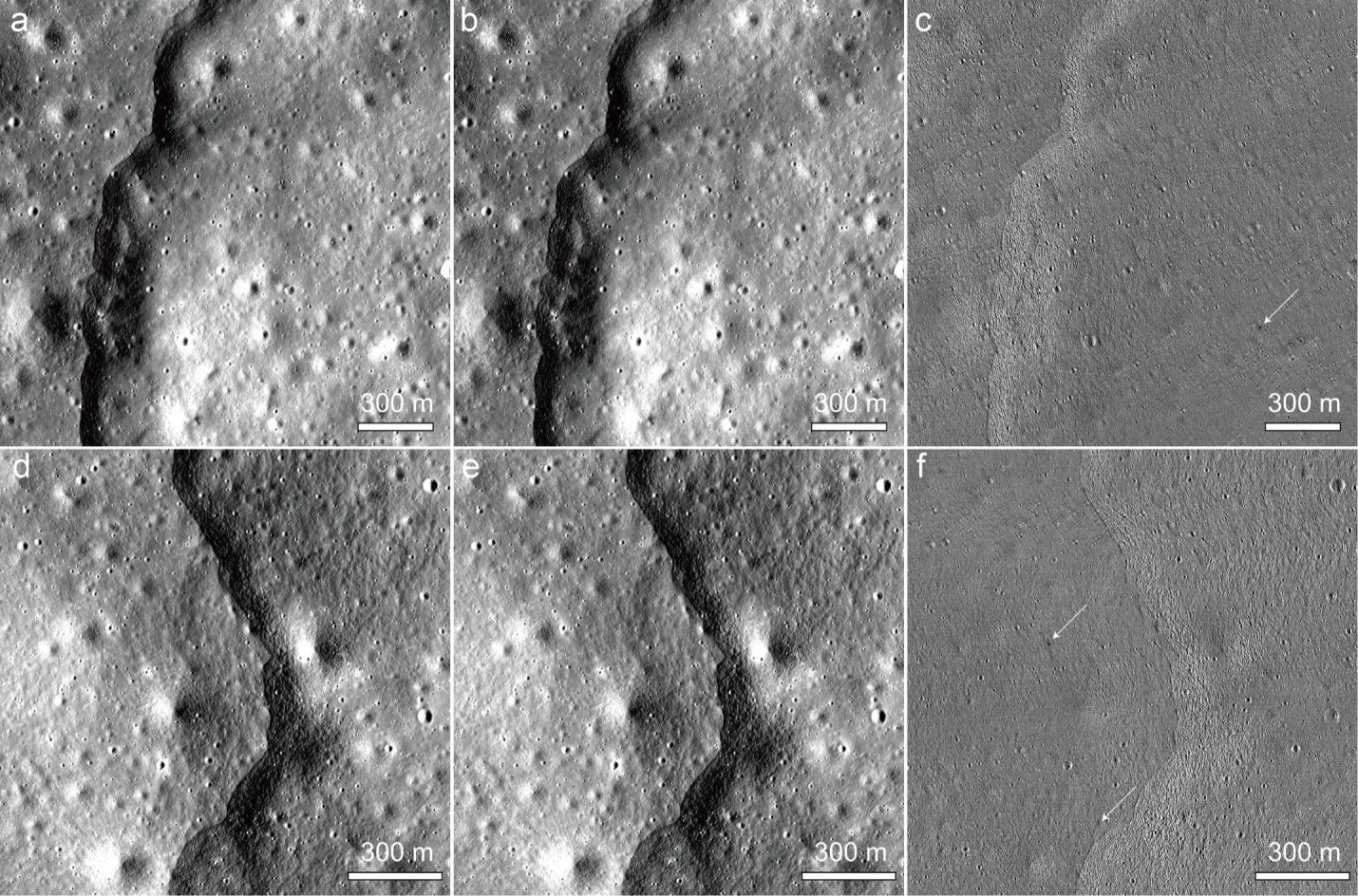


Supplementary Fig. S46. New landslides are not observed along the Mandel'shtam-3 lobate scarp that was interpreted to be possible epicenter of strong shallow moonquakes recorded by Apollo^19^, but new impact splotches are abundant (white arrows). (a–c) Before, after and temporal ratio images, respectively. The new impacts occur as splotches caused by impact rays [8], while the topography of impact craters is not discernible due to their small sizes. The central coordinates of this area are 6.830°N, 161.030°E. IDs and addresses of data used in this figure are available at Table 3.


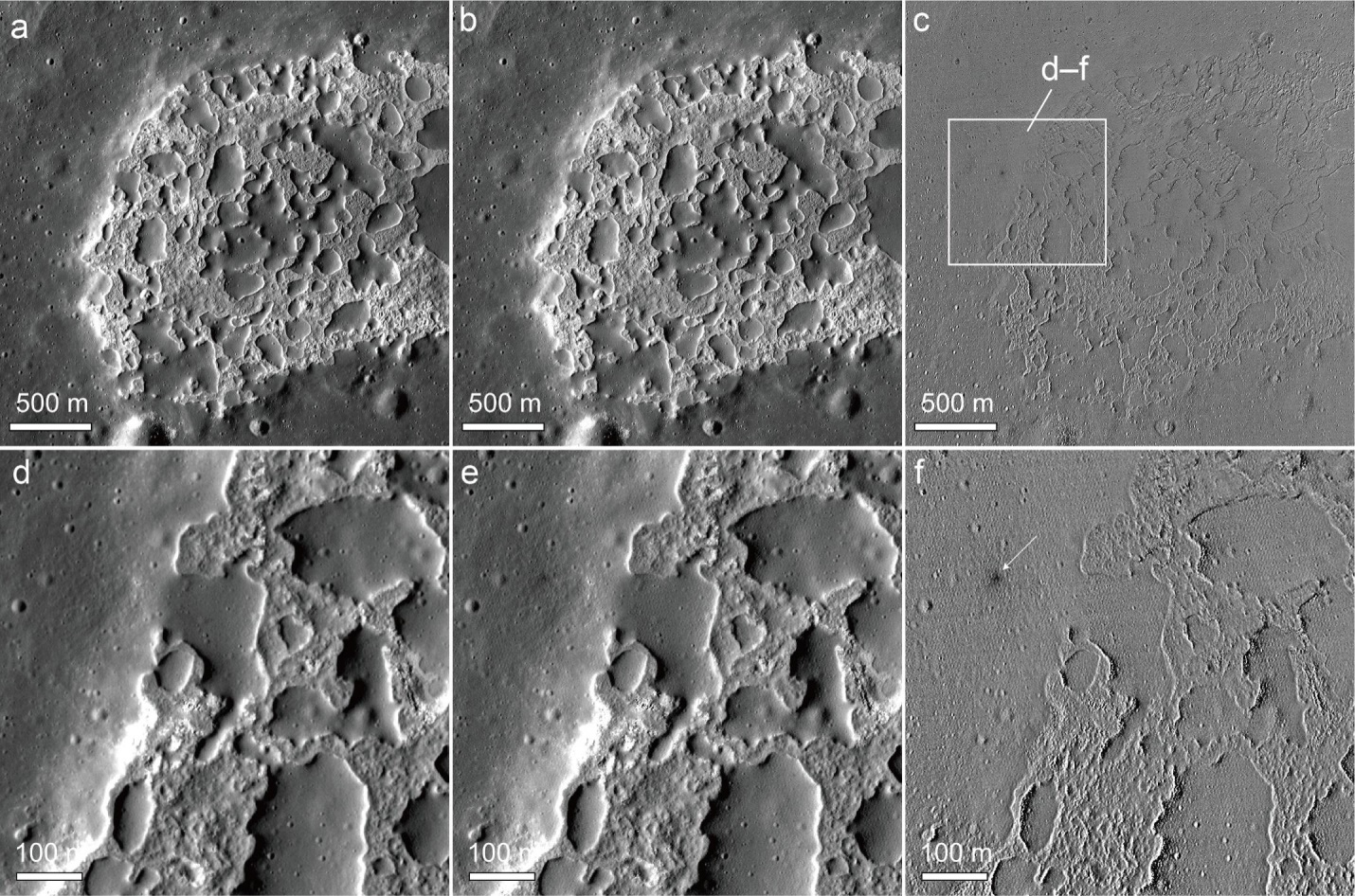


Supplementary Fig. S47. New landslides are not observed in the Ina irregular mare patch (central coordinates 18.65°N, 5.3°E), but a small new impact splotch (white arrow) is visible. (a–c) Before, after and temporal ratio images, respectively. Panels (d–e) are enlarged view for the outlined region shown in panel (c). The new impacts occur as splotches caused by impact rays [8], while the topography of impact craters is not discernible due to their small sizes. IDs and addresses of data used in this figure are available at Table 3.


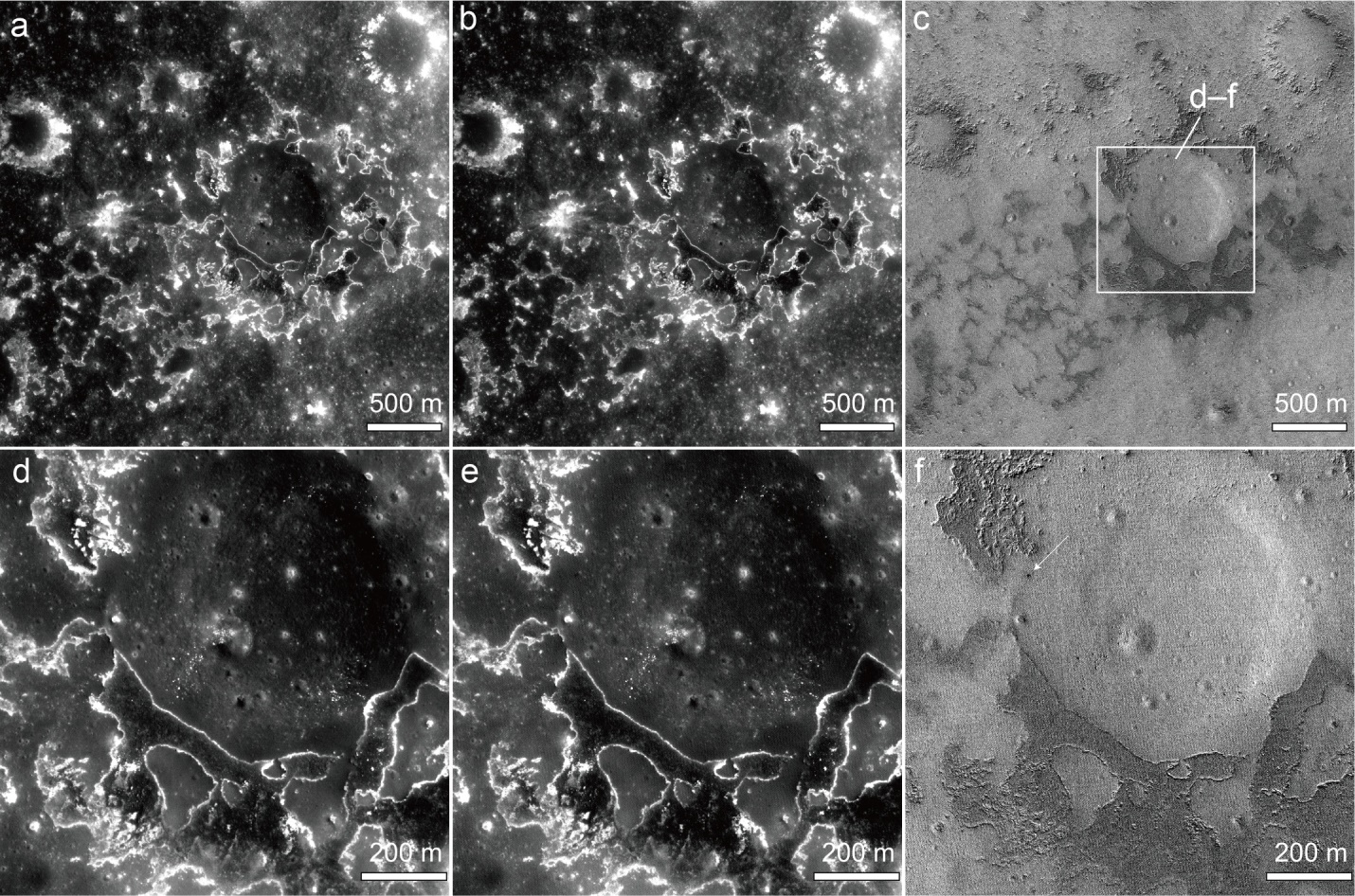


Supplementary Fig. S48. New landslides are not observed in the Maskelyne irregular mare patch (central coordinates 4.33°N, 33.75°E), but a small new impact splotch is visible (white arrow). (a–c) Before, after and temporal ratio images, respectively. Panels (d–e) are enlarged view for the outlined region shown in panel (c). New impact occurs as a splotch caused by impact rays [8], while the topography of impact crater is not discernible due to its small size. IDs and addresses of data used in this figure are available at Table 3.


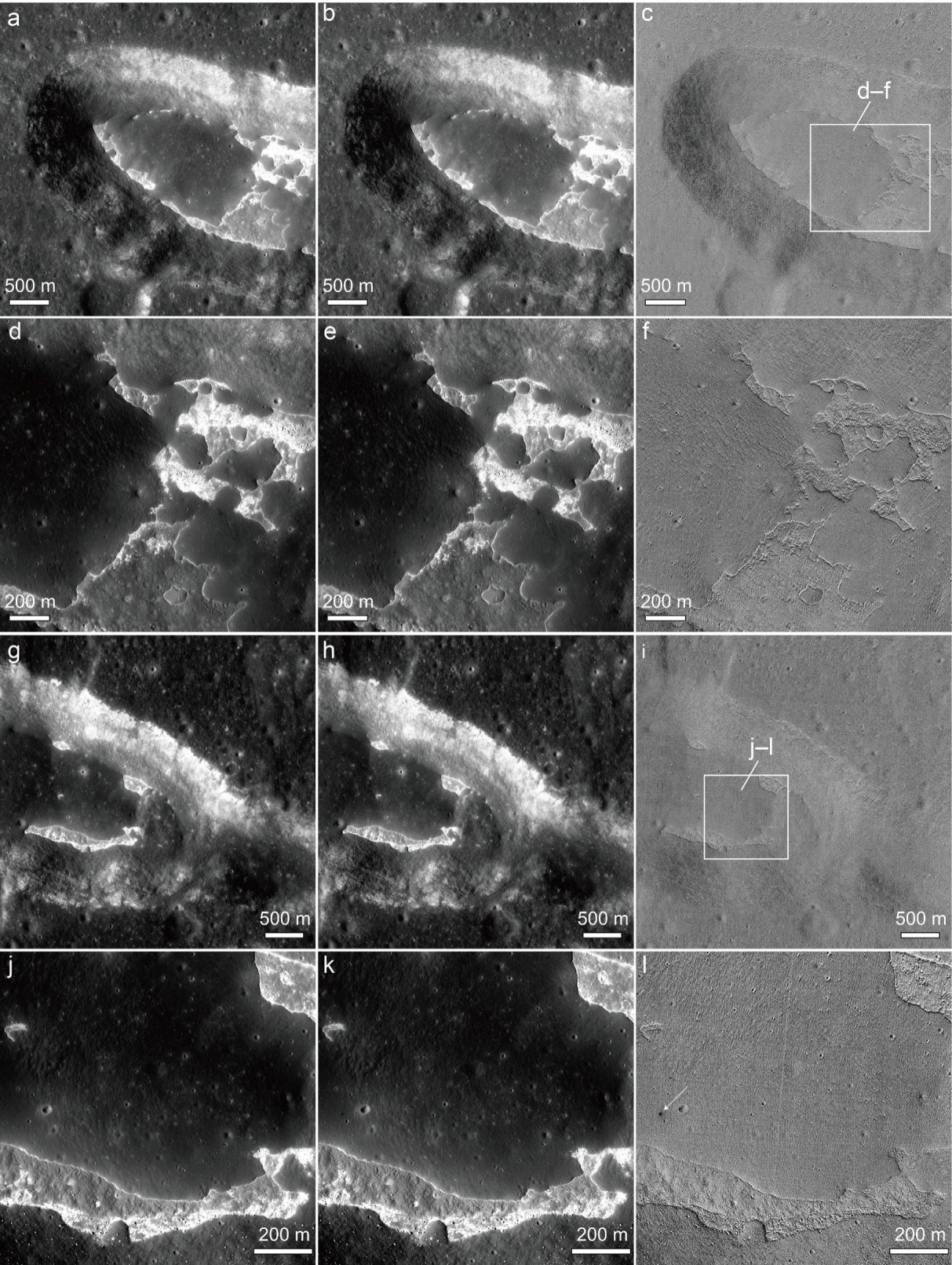


Supplementary Fig. S49. New landslides are not observed in the Sosigenes irregular mare patch (central coordinates of 8.335°N, 19.071°E), but new impact splotch is visible (white arrow). (a–c) and (g–i) are for the western and eastern portion of this irregular mare patch. (d–f) and (j–l) are enlarged views for the outlined zones shown in panels (c) and (i), respectively. The left, middle and right panels are before, after and temporal ratio images, respectively. New impact occurs as a splotch caused by impact rays [8], while the topography of impact crater is not discernible due to its small size. IDs and addresses of data used in this figure are available at Table 3.

Supplementary Table 1. Summary of new landslides that were likely triggered by new impacts. Stratigraphic ages are referred from the 1:5,000,000 unified geological map of the Moon published by the USGS Astrogeology Science Center [35]. Latitudes and longitudes are for the new landslides. The acquisition times of before and after images of temporal pairs that captured the new landslides are shown. Slopes are measured using the SLDEM using a baseline of 60 m.

| No. | Terrain Name | Stratigraphic Age | Latitude (°N) | Longitude (°E) | Time (before) | Time (after) | Slope (°) |
| --- | --- | --- | --- | --- | --- | --- | --- |
| 1 | Aristillus | Eratosthenian | 33.491 | 2.098 | 2013/9/18 | 2023/3/5 | 24 |
| 2 | Briggs B | Copernican | 28.535 | -70.924 | 2013/10/21 | 2014/10/10 | 39 |
| 3 | Copernicus | Copernican | 10.187 | -21.473 | 2014/9/10 | 2015/8/30 | 32 |
| 4 | Delisle | Imbrian | 30.229 | -34.946 | 2013/8/25 | 2019/5/23 | 34 |
| 5 | Diophantus | Nectarian | 27.420 | -34.451 | 2014/11/4 | 2015/10/24 | 31 |
| 6 | Gambart C | Copernican | 3.218 | -11.908 | 2013/11/13 | 2015/10/23 | 32 |
| 7 | Naumann | Imbrian | 35.410 | -62.188 | 2014/5/13 | 2021/12/18 | 36 |
| 8 | Ohm | Eratosthenian | 18.542 | -114.754 | 2018/1/11 | 2020/11/9 | 36 |
| 9 | PicoE | Eratosthenian | 43.104 | -10.334 | 2015/1/10 | 2016/12/18 | 32 |
| 10 | Theaetetu | Eratosthenian | 37.260 | 5.799 | 2015/3/31 | 2021/11/16 | 32 |
| 11 | Timocharis | Eratosthenian | 26.221 | -13.041 | 2014/8/13 | 2019/5/21 | 33 |
| 12 | Tycho | Copernican | -43.338 | -13.025 | 2018//7/26 | 2021/5/25 | 34 |

**Supplementary Table 2. Summary of new landslides triggered by endogenic seismic activity.** Stratigraphic ages are referred from the 1:5,000,000 unified geological map of the Moon published by the USGS Astrogeology Science Center [35]. Latitudes and longitudes are for the new landslides. The acquisition times of before and after images of temporal pairs that captured the new landslides are shown. Slopes are measured using the SLDEM using a baseline of 60 m.

| No. | Terrain Name | Stratigraphic Age | Latitude (°N) | Longitude (°E) | Time (before) | Time (after) | Slope (°) |
| --- | --- | --- | --- | --- | --- | --- | --- |
| 1 | Archimedes | Imbrian | 30.902 | -4.460 | 2013/4/22 | 2023/9/26 | 30 |
| 2 | Aristillus | Eratosthenian | 34.270 | 0.358 | 2015/8/28 | 2019/6/16 | 35 |
| 3 |  |  | 34.232 | 0.388 | 2015/8/28 | 2019/6/16 | 35 |
| 4 |  |  | 34.190 | 0.336 | 2015/8/28 | 2019/6/16 | 33 |
| 5 |  |  | 34.190 | 0.336 | 2015/8/28 | 2019/6/16 | 33 |
| 6 |  |  | 34.013 | 0.331 | 2015/8/28 | 2019/6/16 | 35 |
| 7 |  |  | 34.400 | 0.577 | 2015/8/28 | 2019/6/16 | 33 |
| 8 | AristillusA | Imbrian | 33.595 | 4.526 | 2013/10/15 | 2015/9/24 | 33 |
| 9 | AristillusB | Eratosthenian | 34.774 | -1.816 | 2012/9/2 | 2019/5/20 | 33 |
| 10 | Autolycus | Eratosthenian | 31.308 | 1.360 | 2014/8/12 | 2021/3/30 | 31 |
| 11 |  |  | 31.114 | 1.962 | 2013/3/25 | 2023/8/30 | 33 |
| 12 |  |  | 31.023 | 2.027 | 2013/3/25 | 2023/8/30 | 35 |
| 13 | Briggs B | Copernican | 28.499 | -70.992 | 2013/10/21 | 2014/10/10 | 36 |
| 14 |  |  | 28.519 | -70.973 | 2013/10/21 | 2014/10/10 | 36 |
| 15 | Gambart A | Copernican | 0.840 | -18.690 | 2014/2/17 | 2019/11/15 | 35 |
| 16 |  |  | 0.844 | -18.681 | 2014/2/17 | 2019/11/15 | 37 |
| 17 |  |  | 0.836 | -18.667 | 2014/2/17 | 2019/11/15 | 38 |
| 18 |  |  | 1.053 | -18.617 | 2014/2/17 | 2019/11/15 | 42 |
| 19 | Heis | Imbrian | 32.547 | -32.208 | 2015/9/27 | 2021/5/26 | 38 |
| 20 | Kepler | Copernican | 8.506 | -38.336 | 2012/6/1 | 2014/5/11 | 34 |
| 21 | Kirch | Eratosthenian | 39.220 | -5.804 | 2014/4/12 | 2023/4/3 | 34 |
| 22 | Laplace A | Eratosthenian | 43.834 | -27.033 | 2013/9/21 | 2014/9/10 | 39 |
| 23 | Naumann | Imbrian | 35.391 | -62.185 | 2014/5/13 | 2021/12/18 | 34 |
| 24 |  |  | 35.384 | -62.189 | 2014/5/13 | 2021/12/18 | 33 |
| 25 | Pytheas | Copernican | 20.630 | -20.344 | 2013/2/28 | 2022/9/12 | 34 |
| 26 | Reiner | Eratosthenian | 7.354 | -55.040 | 2017/6/16 | 2020/4/15 | 37 |
| 27 | Ryder | Nectarian | -44.043 | 143.514 | 2016/8/6 | 2020/5/25 | 30 |
| 28 | Tycho | Copernican | -43.346 | -13.034 | 2018//7/26 | 2021/5/25 | 39 |
| 29 |  |  | -44.125 | -12.746 | 2015/4/28 | 2021/12/14 | 35 |

Table 3.Data ID of images used in the main text and supplementary materials. The Kaguya Terrain Camera images used in Fig. S35 are available at https://www.darts.isas.jaxa.jp/planet/pdap/selene/. The LROC NAC images are available at https://www.lroc.asu.edu/.

| Figure number | Data ID | Figure number | Data ID | Figure number | Data ID |
| --- | --- | --- | --- | --- | --- |
| 1a | Lunar_LRO_LROC-WAC_Mosaic_global_100m | | | | |
| 1b | M1287271152LE | 1c | M1376560288LE | 1d | 1c/1b |
| 2a | M1169742753LE | 2b | M1200347023LE | 2c | 2b/2a |
| 2d | M1164952423RE | 2e | M1195558276RE | 2f | 2e/2d |
| 3a | M1376643242LE/M1197976848LE | 3b | M1481045431LE/M1320269943LE | 3c | M1394483018RE/M1154635620RE |
| 3d | M1272304793RE/M1182893161LE | 4a | M1149637354LE/M1119014742LE | 4b–4c | NAC DTM SEPTIMPACT E172S3396 |
| 4d, 4g | M1119014742LE | 4e, 4h | M1149637354LE | 4f | 4e/4d |
| 4i | 4h/4g | | | | |
| Supplementary Figs. S1–S49 | | | | | |
| 1a | M1256610808LE | 1b | M1404615842RE | 1c | 1b/1a |
| 1d | M1165458715LE | 1e | M1316026525RE | 1f | 1e/1d |
| 1g | M1182927579LE | 1h | M1333447900RE | 1i | 1h/1g |
| 1j | M1178417347LE | 1k | M1328956553RE | 1l | 1k/1j |
| 1m | M1119263809RE | 1n | M1448391277RE | 1o | 1n/1m |
| 2a | M1164795427LE | 2b | M1432732655RE | 2c | 2b/2a |
| 3a | M1137010370LE | 3b | M1167624847LE | 3c | 3b/3a |
| 4a | M1132063100LE | 4b | M1313239922RE | 4c | 4b/4a |
| 5a | M1138987659LE | 5b | M1200206882LE | 5c | 5b/5a |
| 6a | M1154635620RE | 6b | M1394483018RE | 6c | 6b/6a |
| 7a | M1270287163RE | 7b | M1359594992RE | 7c | 7b/7a |
| 8a | M1175489735LE | 8b | M1236691253RE | 8c | 8b/8a |
| 9a | M1182445832LE | 9b | M1391690849LE | 9c | 9b/9a |
| 10a | M1162539966RE | 10b | M1313099638RE | 10c | 10b/10a |
| 11a | M1287271152LE | 11b | M1376560288LE | 11c | 11b/11a |
| 12a | M1121273915LE | 12b | M1450399140LE | 12c | 12b/12a |
| 13a | M1195410199RE | 13b | M1315358530RE | 13c | 13b/13a |
| 14a | M1195410199RE | 14b | M1315358530RE | 14c | 14b/14a |
| 15a | M1195410199RE | 15b | M1315358530RE | 15c | 15b/15a |
| 16a | M1195410199LE | 16b | M1315358530LE | 16c | 16b/16a |
| 17a | M1195410199RE | 17b | M1315358530RE | 17c | 17b/17a |
| 18a | M1136519652RE | 18b | M1197737691RE | 18c | 18b/18a |
| 19a | M1101216281LE | 19b | M1313022267LE | 19c | 19b/19a |
| 20a | M1118873621LE | 20b | M1448012537LE | 20c | 20b/20a |
| 20d | M1118873621LE | 20e | M1448012537LE | 20f | 20e/20d |
| 21a | M1162447339RE | 21b | M1371731967RE | 21c | 21b/21a |
| 22a | M1137010370LE | 22b | M1167624847LE | 22c | 22b/22a |
| 23a | M1137010370LE | 23b | M1167624847LE | 23c | 23b/23a |
| 24a | M1147274974RE | 24b | M1328420891RE | 24c | 24b/24a |
| 25a | M1147274974LE | 25b | M1328420891LE | 25c | 25b/25a |
| 26a | M1197976848LE | 26b | M1376643242LE | 26c | 26b/26a |
| 27a | M193210868RE | 27b | M1154471358LE | 27c | 27b/27a |
| 28a | M1151909348RE | 28b | M1435124641LE | 28c | 28b/28a |
| 29a | M1134365669LE | 29b | M1164980196LE | 29c | 29b/29a |
| 30a | M1154635620RE | 30b | M1394483018RE | 30c | 30b/30a |
| 31a | M1116664315RE | 31b | M1417663687RE | 31c | 31b/31a |
| 32a | M1252250955LE | 32b | M1341576286RE | 32c | 32b/32a |
| 33a | M1225097473RE | 33b | M1345006352RE | 33c | 33b/33a |
| 34a | M1184894296LE | 34b | M1394108839LE | 34c | 34b/34a |
| 35 | TCO_MAPm04_N33E000N30E003C, TCO_MAPm04_N33E003N30E006C  TCO_MAPm04_N33E357N30E360C, TCO_MAPm04_N36E000N33E003C  TCO_MAPm04_N36E003N33E006C, TCO_MAPm04_N36E357N33E360C  TCO_MAPm04_N39E000N36E003C, TCO_MAPm04_N39E003N36E006C  TCO_MAPm04_N39E357N36E360C | | | | |
| 36a | M1279519787RE | 36b | M1368815816RE | 36c | 36b/36a |
| 37a | M1253256935LE | 37b | M1342581797RE | 37c | 37b/37a |
| 38a | M1370952171LE | 38b | M1429617636LE | 38c | 38b/38a |
| 39a | M1193246004R | 39b | M1223844488LE. | 39c | 39b/39a |
| 40a | M1219526641LE | 40b | M1339438884LE | 40c | 40b/40a |
| 41a | M1236851704LE | 41b | M1267424171RE | 41c | 41b/41a |
| 42a | M1256610808LE | 42b | M1404615842RE | 42c | 42b/42a |
| 43a | M1256610808LE | 43b | M1404615842RE | 43c | 43b/43a |
| 44a | M1257223037LE | 44b | M1405233637LE | 44c | 44b/44a |
| 45a | M1173705714L | 45b | M1204310939LE | 45c | 45b/45a |
| 46a | M1107224682LE | 46b | M1199074484LE | 46c | 46b/46a |
| 46d | M1107224682LE | 46e | M1199074484LE | 46f | 46e/46d |
| 47a | M1138873574RE | 47b | M1437412933LE | 47c | 47b/47a |
| 47d | M1138873574RE | 47e | M1437412933LE | 47f | 47e/47d |
| 48a | M1223443814LE | 48b | M1343347623LE | 48c | 48b/48a |
| 48d | M1223443814LE | 48e | M1343347623LE | 48f | 48e/48d |
| 49a | M1114042961LE | 49b | M1443195256LE | 49c | 49b/49a |
| 49d | M1114042961LE | 49e | M1443195256LE | 49c | 49e/49d |
| 49g | M1114042961RE | 49h | M1443195256RE | 49i | 49h/49g |
| 49j | M1114042961RE | 49k | M1443195256RE | 49l | 49k/49j |
